# The GUIDES checklist: development of a tool to improve the successful use of guideline-based computerised clinical decision support

**DOI:** 10.1186/s13012-018-0772-3

**Published:** 2018-06-25

**Authors:** Stijn Van de Velde, Ilkka Kunnamo, Pavel Roshanov, Tiina Kortteisto, Bert Aertgeerts, Per Olav Vandvik, Signe Flottorp, GUIDES expert panel, Smisha Agarwal, Leila Ahmadian, David Bates, Linn Brandt, Romina Brignardello-Petersen, Carl Cauwenbergh, Yaolong Chen, Nicholas Conway, Nicolas Delvaux, Pierre Durieux, Robert El-Kareh, Atle Fretheim, Robert Greenes, Robert Brian Haynes, Annemie Heselmans, Tim Holt, Robert A. Jenders, Kensaku Kawamoto, Tamara Kredo, Edwin Lomotan, Marjolein Lugtenberg, Luis Marco-Ruiz, Colin McCowan, Lisa McDermott, Stephanie Medlock, Maria Michaels, Blackford Middleton, Marc Mitchell, Lorenzo Moja, Michael Mugisha, Jerome A. Osheroff, Pablo Alonso Coello, Sallie-Anne Pearson, Sylvia Pelayo, Joshua Richardson, Peeter Ross, Nard Schreurs, Matthew Semler, Dean Sittig, Tigest Tamrat, Madis Tiik, Anna Turusheva, Heleen van der Sijs, Robert Vander Stichele, Mieke Vermandere, Abigail Viall, Mahima Venkateswaran, Adam Wright, Taryn Young

**Affiliations:** 1 Centre for Informed Health Choices, Division for Health Services, Norwegian Institute of Public Health, Oslo, Norway; 2 Duodecim, Scientific Society of Finnish Physicians, Helsinki, Finland; 3 Department of Medicine, McMaster University, Hamilton, Canada; 4 Tampere University Hospital, Tampere, Finland; 5 Department of Public Health and Primary Care, KU Leuven, Leuven, Belgium; 6 MAGIC Non-Profit Research and Innovation Programme, Oslo, Norway; 7 Institute of Health and Society, University of Oslo, Oslo, Norway

**Keywords:** Clinical computerised decision support systems, Practice Guidelines, Guideline adherence, Evidence-based medicine, Implementation

## Abstract

**Background:**

Computerised decision support (CDS) based on trustworthy clinical guidelines is a key component of a learning healthcare system. Research shows that the effectiveness of CDS is mixed.

Multifaceted context, system, recommendation and implementation factors may potentially affect the success of CDS interventions. This paper describes the development of a checklist that is intended to support professionals to implement CDS successfully.

**Methods:**

We developed the checklist through an iterative process that involved a systematic review of evidence and frameworks, a synthesis of the success factors identified in the review, feedback from an international expert panel that evaluated the checklist in relation to a list of desirable framework attributes, consultations with patients and healthcare consumers and pilot testing of the checklist.

**Results:**

We screened 5347 papers and selected 71 papers with relevant information on success factors for guideline-based CDS. From the selected papers, we developed a 16-factor checklist that is divided in four domains, i.e. the CDS context, content, system and implementation domains. The panel of experts evaluated the checklist positively as an instrument that could support people implementing guideline-based CDS across a wide range of settings globally. Patients and healthcare consumers identified guideline-based CDS as an important quality improvement intervention and perceived the GUIDES checklist as a suitable and useful strategy.

**Conclusions:**

The GUIDES checklist can support professionals in considering the factors that affect the success of CDS interventions. It may facilitate a deeper and more accurate understanding of the factors shaping CDS effectiveness. Relying on a structured approach may prevent that important factors are missed.

**Electronic supplementary material:**

The online version of this article (10.1186/s13012-018-0772-3) contains supplementary material, which is available to authorized users.

## Background

Increasing the value and reducing waste in healthcare are important issues within resource-constrained systems globally [1]. Both the underuse and overuse of healthcare services can have serious consequences for people’s health and for healthcare spending [2–4]. The most important drivers of poor care fall into three domains: money and finance; knowledge, bias and uncertainty; and power and human relationships [5]. Computerised decision support (CDS) based on trustworthy clinical guidelines can play a key role in addressing some of these problems by improving knowledge, accelerating the adoption of new evidence and helping to better manage beliefs, assumptions and uncertainty among healthcare providers and patients [3, 5]. The US National Academy of Medicine (formerly the Institute of Medicine) has identified CDS as a key component of a learning healthcare system [6].

CDS is a technology that provides patient-specific medical knowledge at the point of need. Research into guideline-based CDS over the last 40 years has shown that the effectiveness and success of CDS have been mixed [7]. Systematic reviews regarding CDS effectiveness estimate on average modest increases in guideline adherence and modest reductions in morbidity [8, 9]. CDS does obviously not work in isolation but it is part of a complicated mingling of determinants. Variations in success may be due to problems with the CDS system, the CDS content, and how the CDS is implemented or because CDS may not be able to provide a complete solution to complex problems [10]. Any decision to use CDS, or other additional interventions, should be based on an assessment of the determinants of healthcare practice that affect whether the desired changes can be achieved [11]. Multiple reviews have evaluated determinants of successful development and use of CDS, for example implementation of CDS in inpatient versus outpatient settings, CDS aimed at prevention or disease management, integration of CDS in charting or order entry system and providing CDS to both clinicians and patients [12–15]. An understanding of which factors or combinations of factors make CDS more or less effective is still being developed [12, 16].

The aim of the GUIDES project was to improve the successful use of guideline-based CDS through the development of a checklist. The purpose of the checklist was to facilitate a deeper and more accurate understanding of which factors make CDS more (or less) effective and to guide CDS implementation by preventing key factors from being overlooked.

## Methods

We developed the checklist in the following steps, as previously published in a detailed protocol for the GUIDES project [17]: (1) a systematic review of the research evidence and frameworks on the factors affecting success of guideline-based CDS, (2) a synthesis of the factors identified and creation of the checklist and (3) pilot testing the checklist by testing it in a systematic review of trials on CDS and by using it during focus group discussions about CDS success features. Currently, there is no standardised methodology on how to develop a checklist. However, the steps included in this process allow broad input from diverse sources regarding checklist content and design which matches with the available guidance for checklist development [18, 19].

Step 1: We (SV and SF) systematically reviewed frameworks, systematic reviews, process evaluations and qualitative evidence pertaining to factors for successful CDS implementation according to a predefined protocol. In an additional review, we focussed on head-to-head trials evaluating success factors of interventions with CDS.

Step 2: We extracted all the identified factors in the selected papers to develop the first version of the GUIDES checklist. An international group of experts with experience in CDS and/or guidelines provided input and feedback in two rounds of consultation and one approval round. To compose the expert group, we invited first the corresponding authors of every relevant paper found in the review phase and combined this with the suggestions by the members of the GUIDES project in order to achieve a balanced group with respect to multidisciplinary representation and international representation. The experts gave their feedback through a structured online feedback form that related to a list of desirable framework attributes. We also collected the views of patients and health care consumers in one round through an additional survey to ensure that the adopted strategies are relevant and acceptable to patients. The detailed questions for each survey are available in Additional files 1 and 2. The group of authors discussed the feedback from every consultation round and agreed on the revisions that we made. We also asked the panel of experts to judge if the included factors were ‘always’ , ‘sometimes’ or ‘never important’ for the success of guideline-based CDS interventions. Further, we asked the experts to select what they regarded as the five most important factors and to rank their selection from the most important to the fifth most important. In parallel with the checklist, we also asked feedback from the expert panel on three support worksheets to help users to (1) select the most important recommendations for implementation, (2) evaluate which implementation strategies are appropriate for the prioritised recommendations and (3) assess if CDS is appropriate for the selected recommendations.

Step 3: We invited six colleagues with research backgrounds to pilot the GUIDES checklist by rating a random sample of 30 trial reports that we identified during our review of head-to-head trials. We asked the members of this group to think aloud as they applied the checklist for the first time, and we made notes about how they interacted with the checklist and how they interpreted the different checklist factors. Two participants independently assessed each trial report, to make judgements about how well the CDS interventions performed in relation to the GUIDES factors for successful CDS. We calculated interrater agreement to assess consistency of rating across the researchers.

We further piloted the checklist during six focus groups with general practitioners and patients who discussed a CDS intervention intended to improve care and outcomes for patients with knee osteoarthritis. In the focus group, participants discussed the factors that determine successful use of CDS during an initial brainstorming phase. In a subsequent structured phase, the moderator used the GUIDES checklist to ask probing questions on factors that were not yet discussed. At the end of the interview, the participants individually selected five factors that they considered the most important for the successful use of the CDS strategy. We evaluated to what extent the checklist allowed us to identify additional factors and how many of these factors were rated as important by the participants. The focus groups took place in Norway, Belgium and Finland. The moderators emphasised that both positive and negative feedback about the CDS intervention was important. We audio-recorded each focus group and an observer took notes. We transcribed key parts of the focus groups but we did not do a full transcription of the recordings. Further details are available in a separate report [20]. Here we reported the findings of an evaluation of the process we used to identify factors that could affect the success of the suggested strategy.

## Results

### Characteristics of the expert panel

A panel of 49 experts provided online feedback to the GUIDES project. Twenty-two of the 49 experts had a background in evidence-based guidelines, 12 were healthcare providers and 42 had a background in CDS. Eight experts were active in CDS system development, implementation and evaluation. Twelve were involved in education, and four had governmental backgrounds, either as CDS programme funders or as health policy analysts. The majority of the experts (84%) had a research background. Seven participants had no experience of CDS. The areas of expertise are based on self-report by the panel members.

The panel consisted of experts from 18 countries across five continents. Ten experts had CDS experience in low- and middle-income countries. Eight reported financial relationships with entities in the health informatics arena; three reported other relationships or activities that may have been potential conflicts of interest. In both feedback rounds, the proportion of participants who responded was above 90%. Four patients and healthcare consumers also provided feedback through an additional survey.

### Development of the checklist

**Fig. 1:**
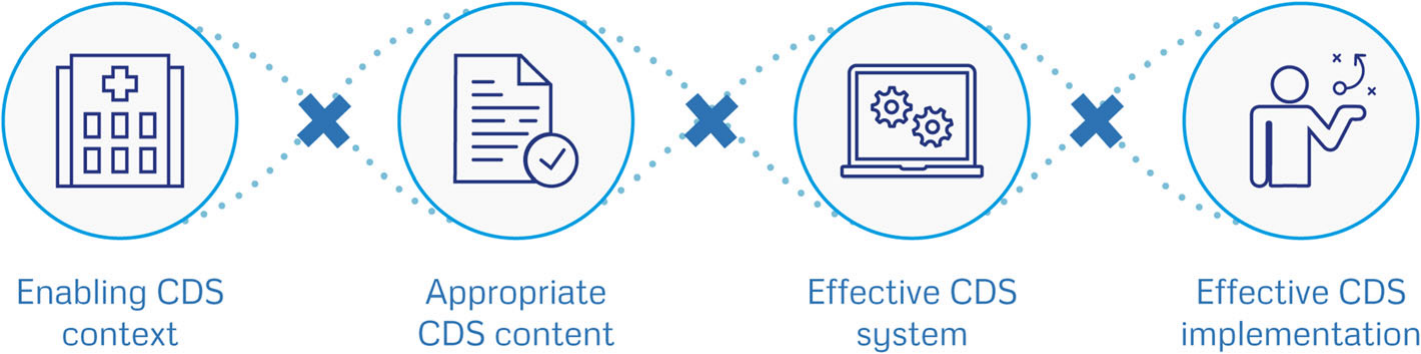
Diagram presenting the four domains that are important for successful implementation of guideline-based CDS. This diagram is adapted from the formula by Fixsen on successful uses of evidence-based programs in human service settings [100]

**Fig. 2:**
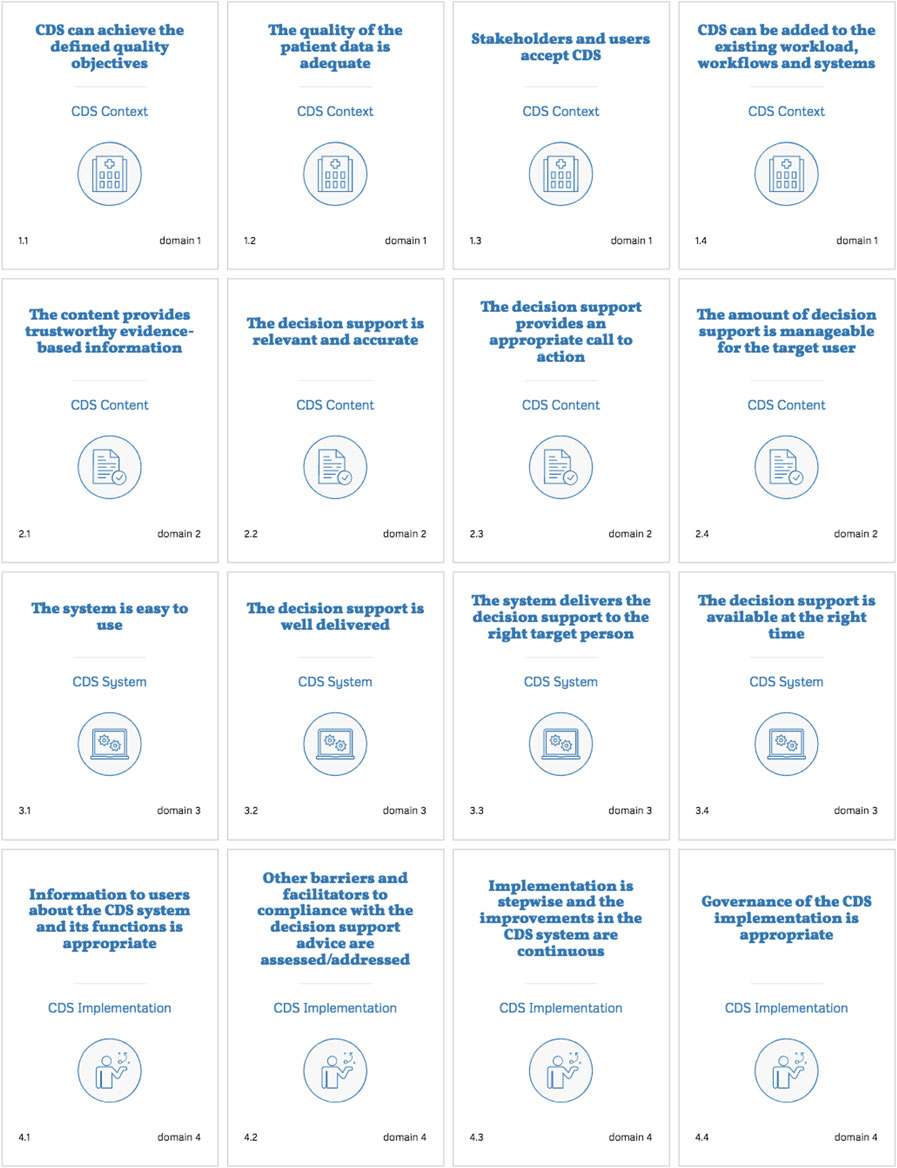
Screenshot of the electronic version of the GUIDES checklist illustrating the domains and factors

Here we describe the results of the literature review, the consultation rounds and the pilot testing. Tables 1 and 2, Figs. 1 and 2 and Additional file 3 provide further details about the content of the GUIDES checklist we developed.

Step 1: Review of the research evidence and frameworks

**Table 1:** An overview of the GUIDES checklist

The checklist contains four CDS domains (Fig. 1). Each domain includes four factors, making 16 factors in total (see Fig. 2). Checklist domains: • Context: the circumstances in which CDS can be potentially successful. • Content: the factors shaping the success of the advice produced by the CDS system. • System: features belonging to the CDS tool. • Implementation: factors affecting the integration of CDS into practice settings. The full checklist (see Additional file 3) provides: • A rationale for the importance of each factor. • Sample questions to consider. • Positive and negative examples. The GUIDES checklist is available in different formats, including an electronic version that enables CDS implementation teams to complete the GUIDES checklist efficiently in a group (see https://www.guidesproject.org).

**Table 2:** Overview of the GUIDES checklist factors and how to evaluate questions

Question	Strongly disagree	Disagree	Somewhat disagree	Undecided	Somewhat agree	Agree	Strongly agree
Domain 1: The CDS context							
1.1. CDS can achieve the planned quality objectives - Does CDS address the factors that explain the current behaviour of healthcare providers and patients? - Does the available evidence support the use of CDS for the given outcomes, tasks and settings?	□	□	□	□	□	□	□
1.2 The quality of the patient data is sufficient - Is the structured patient data that is needed to achieve the CDS objective sufficiently accurate and complete to allow the use of CDS? - If necessary, can the quality of the data be improved or can the CDS itself improve the data quality?	□	□	□	□	□	□	□
1.3 Stakeholders and users accept CDS - Is there a clear benefit to the users who will engage with the CDS? - Do the users and stakeholders have a positive attitude towards the use of CDS? - If necessary, is it possible to increase user and stakeholder acceptance?	□	□	□	□	□	□	□
1.4 CDS can be added to the existing workload, workflows and systems - Is the required hardware available and what will the impact be of adding CDS to the existing information systems? - Is it feasible to introduce CDS, given the current workload and the usual work processes? - If necessary, can the workload or the work processes be changed or can the CDS system improve the workload or work processes?	□	□	□	□	□	□	□
Domain 2: The CDS content							
2.1 The content provides trustworthy evidence-based information - Do the organisation(s) and people that developed the decision support have credibility? - Is the advice supported by up-to-date scientific evidence and is the type and quality of this evidence clear to the user? - Is the decision support clear on the benefits and harms of the different management options?	□	□	□	□	□	□	□
2.2 The decision support is relevant and accurate - Does the decision support contain accurate information that is pertinent to the care of the patient? - Does the decision support address the information needs of the users? - Is it clear to the users why the decision support information is provided for a given patient?	□	□	□	□	□	□	□
2.3 The decision support provides an appropriate call to action - Is the recommended action clear enough for the targeted users to act on? - Is the clinical importance and urgency of the recommended action sufficiently clear? - Is the advice applicable in the setting in which it will be implemented? - Is it clear how the recommended action fits with other current guidelines?	□	□	□	□	□	□	□
2.4 The amount of decision support is manageable for the target user - Is the total amount of decision support manageable for the healthcare provider? - Is the amount of decision support per patient manageable?	□	□	□	□	□	□	□
Domain 3: The CDS system							
3.1 The system is easy to use - Is it easy for users to interact with the CDS system? - Does the system facilitate (or, at least, not hinder) the workflow of the healthcare providers? - Can the system be customised to provide better user support - Is the system always up and running?	□	□	□	□	□	□	□
3.2 The decision support is well delivered - Is the advice delivered in an appropriate mode, format and channel? - Is the display of the decision support eye-catching, intuitive, concise, consistent and unambiguous? - Is it appropriate to use specific functions (e.g. pop-ups, computerised restrictions, indications of (dis)agreement) for prioritised decision support?	□	□	□	□	□	□	□
3.3 The system delivers the decision support to the right target person - Is the system reaching the targeted users (healthcare providers and/or patients)? - Is the system able to facilitate team processes when these are needed?	□	□	□	□	□	□	□
3.4 The decision support is available at the right time - Does the system provide the decision support at a moment of need?	□	□	□	□	□	□	□
Domain 4: The CDS implementation							
4.1 Information to users about the CDS and its functions is appropriate - Is the communication and documentation about the CDS appropriate? - Are help topics related to the functioning of the CDS system available to users? - If necessary, is user training available?	□	□	□	□	□	□	□
4.2 Other barriers and facilitators to compliance with the decision support advice are assessed/addressed - Is there an assessment of the beliefs, attitudes and skills of the healthcare providers and patients that may affect adherence to the decision support advice? Are actions planned/taken accordingly? - Is there an assessment of the professional interactions affecting adherence to the recommended actions, and are actions planned/taken accordingly? - Is there an assessment of the (dis)incentives affecting the adherence of healthcare providers and patients to the decision support advice? Are necessary actions planned/taken? - Is there an assessment of the issues related to the capacity and resources needed to ensure adherence to the recommended actions? Are the necessary actions planned/taken? - Does the organisational context influence adherence to the decision support advice and what are actions planned/taken accordingly?	□	□	□	□	□	□	□
4.3 Implementation is stepwise and the improvements in the CDS system are continuous - Is the implementation of the CDS stepwise? - Is a plan in place to collect user feedback and to monitor system usage, performance and outcomes? - Are malfunctions and other problems with use of the CDS quickly fixed?	□	□	□	□	□	□	□
4.4 Governance of the CDS implementation is appropriate - Are all the key stakeholders involved in the planning and implementation of the system? - Is the CDS initiative governed in an efficient, sustainable and equitable way?	□	□	□	□	□	□	□

The search identified 5347 papers. The final selection included 71 papers (including 21 frameworks, 16 systematic reviews, 7 qualitative studies and 27 process evaluations related to CDS trials). We excluded several other systematic reviews because their scope was too narrow, focusing for example on a specific clinical condition, setting or CDS function. We double-checked that no information was lost by excluding these reviews. We also excluded some older systematic reviews because more recent reviews of the same research question were available. We double-checked that no information was lost by excluding these reviews.

The included frameworks most frequently discussed items related to the ease of use of CDS systems, stepwise implementation of CDS interventions, continuous improvements of CDS systems, CDS delivery methods, relevance and accuracy of CDS and the trustworthiness of CDS content [21–41]. The frameworks were often rooted in the experiences of particular national or regional settings. Fifteen frameworks were developed in the USA, five in Europe and one in Australia. None of the included frameworks met all the preferred framework attributes. Many frameworks focussed on a specific clinical setting, type of practice or type of CDS function. The diverse goals of the frameworks made it difficult to evaluate their usability. Most of the frameworks were presented in research papers which detailed CDS classifications, the lessons learned or recommendations. In only two instances was it explicitly mentioned that CDS developers and/or implementers were the primary target audience of the frameworks.

The systematic reviews evaluated a wide range of aspects related to the success of CDS interventions [12, 42–56]. The reviews focussed most on the role of the CDS setting and target, the methods to deliver the CDS and the trustworthiness of the decision support. In a separate publication, we discuss these results in relation to the findings of another systematic review that we conducted (*Unpublished research submitted to Implementation Science*). That report also rates the certainty of the evidence for every factor using the GRADE approach [57].

The qualitative studies and process evaluations that we included often discussed issues related to the acceptance of CDS, the ease of use of a CDS system and the role of other factors influencing compliance with decision support advice [58–91].

Step 2: Synthesis of CDS success factors and development of GUIDES checklist

From the selected papers, we extracted 816 excerpts that were related to factors affecting CDS success. We coded all the information contained in the excerpts and used this to construct a first checklist version. GUIDES checklist v1.1 included 38 items classified in four domains, namely, the CDS context, content, system and implementation. The expert panel provided input on this draft checklist, and we made a number of important adjustments.

In the GUIDES checklist v1.2, we reduced the number of factors to 19 by grouping items under factors representing ‘higher-level’ concepts. No items were removed. For each factor, we included a set of questions to help users in their evaluation. Examples of positive and negative impacts were included. Items that were complicated, unclear or ambiguous were reworded.

The expert feedback on v1.2 was positive overall. Eighty-nine percent of the experts judged the checklist to be an appropriate tool for helping to identify factors that should be considered when implementing guideline-based CDS. The remaining 11% of experts were not able to judge this or commented on the limitation that the checklist does not suggest potential solutions for the problems identified or the limitation that the checklist does not address the implementability of guidelines. The experts agreed that the checklist would be applicable across different settings and different types of practices (82%). The remaining experts were uncertain as to how the checklist would perform for the implementation of CDS in small practices, in tertiary care and in low- and middle-income countries. The experts found that the checklist was logically organised in a way that it was easy to understand (93%) and that the factors were labelled and explained in ways that were easy to understand (84%). Twenty-nine percent of the experts indicated that they felt that parts of the checklist were too complicated. Sixty-four percent of the experts found that no potentially important factors were missing. The other experts either found that some descriptions were implicit or suggested to discuss various concepts such as equitable implementation of CDS, patient involvement or use of CDS on handheld devices. Eighty-four percent concluded that the checklist did not include irrelevant factors. Some other experts pointed at interrelations or overlap between the factors. The panel noted that the checklist was suitable for people with a research background (80%). Other experts suggested further adjustments or asked data about how well the checklist achieves its purpose. Forty-two percent observed that pilot testing would be required to evaluate if the checklist is suitable for use by people who are not researchers. Ninety percent of the experts indicated that they intend to use the checklist. Other experts were uncertain, and one expert mentioned not to need the checklist given the personal expertise acquired by many CDS implementations.

Additional file 1 provides more details from the expert panel feedback.

In the GUIDES checklist v1.3, we regrouped the checklist to a list of 16 factors and we made other adjustments as a response to the expert feedback. We presented the checklist once more to the panel, which approved it unanimously. Table 1 provides a short overview of the checklist and the full checklist is available in Additional file 3. The response of the expert panel to the three additional worksheets was mixed. Therefore, we decided not to include these with the GUIDES checklist. However, they are available on request.

Patients and healthcare consumers strongly agreed that guideline-based CDS is an important quality improvement intervention (100%) and that the GUIDES checklist is a suitable (75%) and potentially beneficial tool (100%). They agreed that there is a need to provide CDS on treatment options to both healthcare providers and patients (sometimes simultaneously for shared decision making). Additional file 2 provides more detailed results for this survey.

Step 3: Pilot testing the GUIDES checklist

Pilot testing the checklist on a sample of CDS trial reports showed that the participants took 60–90 min to complete the checklist. We noted and adjusted some redundancies across the checklist factors. Interrater agreement was generally poor, with kappa values lower than 0.60. The raters often found the quality of the reporting and information about the trials to be ambiguous or unclear. This led to notable variations between the evaluations. After the initial ratings were completed, the six raters resolved disagreements by discussion. They agreed that there was insufficient information to make judgements about the quality of a CDS intervention for half of the trial reports. Another frequent cause of disagreement related to some raters having overlooked specific information available in the trial reports. In other instances, raters made different judgements in relation to specific factors. The pilot testing indicated that the GUIDES checklist could be applied to a range of different settings and could be used to classify all the descriptive information on interventions and contexts.

Eight healthcare providers and 22 patients participated in the focus group interviews. The focus groups yielded 211 factors potentially affecting CDS success. After combining related factors, we identified 59 unique factors. All the suggested factors could be classified under the GUIDES checklist factors. Half of the suggested factors were related to five checklist items: 1.1 ‘CDS can achieve the planned quality objectives’ , 1.2 ‘The quality of the patient data is sufficient’ , 1.4 ‘CDS can be added to the existing workload, workflows and systems’ , 2.2 ‘The content is relevant and accurate’ and 3.2 ‘The decision support is well delivered’. The participants suggested a median of 17 unique factors during the brainstorm phase and 13 additional factors during the structured phase when the GUIDES checklist was used. Per focus group, the participants prioritised a median of eight factors that were suggested during the brainstorming phase and a median of six factors that were suggested during the structured phase. Overall, ten factors were suggested only during the structured interview phase, out of which six were prioritised.

### The most important GUIDES checklist factors

The panel indicated that all the factors we had included were important and that a failure to consider all of them could potentially cause a CDS intervention to fail. They noted, however, that the level of importance of each factor might vary across circumstances and settings. For example, 30% of the experts indicated that factors in the CDS implementation domain were only sometimes important.

The survey of the expert panel showed that they considered factor 2.1 ‘The content provides trustworthy evidence-based information’ to be the most important factor. Other factors that scored high were 2.2 ‘The decision support is relevant and accurate’ , 1.4 ‘CDS can be added to the existing workload, workflows and systems’ and 1.1 ‘CDS can achieve the defined quality objectives’.

Additional file 4 provides more detailed results on the importance related ratings by the expert panel.

## Discussion

The goal of the GUIDES project was to increase the success of guideline-based CDS. By developing a checklist, we aimed to assist those involved with the implementation of CDS interventions to consider success factors for guideline-based CDS in a structured way. When designing the checklist, our biggest challenge was to make it comprehensive and, at the same time, concise and easy to apply. The expert panel agreed that the final checklist would be potentially beneficial to people implementing guideline-based CDS across a wide range of settings globally. Ninety percent of study participants indicated that they intend to use the checklist.

### Relation to findings from studies in related fields

We identified two studies in related fields. Ross et al. conducted an overview of reviews focusing on factors affecting the implementation of e-health in general [92]. The review did not include any factors beyond those found in the GUIDES checklist. Wu et al. conducted an interdisciplinary systematic review and identified factors shaping the success of CDS interventions which are generalisable across healthcare and non-healthcare fields (for example, defence, finance, aviation and the environment) [93]. The seven features they listed correspond closely with the factors included in the GUIDES checklist.

### Limitations and perspectives

The checklist may have design shortcomings because it has not been extensively tested with people who are not researchers. While the majority of the panel members was involved with research, more than a third was active with both research and clinical practice or implementation work. We plan to evaluate the experiences of users in the diverse project settings in which the GUIDES checklist will be used. The electronic GUIDES platform will also help us to collect user feedback systematically. A first test case includes the ELMO trial, where GUIDES was used to plan the implementation strategy for CDS aimed to improve the appropriateness of diagnostic testing [94]. We recognise that some checklist users may need training in how to use it, and we encourage people to contact us for assistance.

In most instances, it is clear why each factor has been assigned to a particular domain. We recognise that in a few instances factors could be assigned to more than one domain (e.g. the factor ‘The system delivers the decision support to the right target person’ has a relation to both the CDS system domain and the implementation domain). Some factors are also interrelated, such as ‘Stakeholders and users accept CDS’, which is also affected by factors in the content, system and implementation domain. Other potential limitations of our checklist are that it does not include strategies to mitigate risks for CDS interventions or to solve problems identified when applying the checklist. For example, the checklist does not address the requirements for guideline design and authoring to facilitate use of guidelines within CDS [95]. We recognise that it would be useful to add information on mitigation strategies in future updates of the GUIDES checklist. Information on strategies to evaluate the success of CDS could also be added in an update. Although the checklist provides a comprehensive overview of the factors affecting the success of CDS interventions, it does not define the minimum requirements for every factor. How, for example, can one determine when a CDS intervention is sufficiently relevant and accurate? What amount of decision support is appropriate? Providing comprehensive detail is challenging given the large variation in CDS contexts and systems, and using the checklist requires careful judgement. Similar limitations have also been identified in other checklist projects for complex interventions [96].

Members of the expert panel gave broad support for the GUIDES checklist. However, we do not yet have evidence that the use of the GUIDES checklist will increase the success of guideline-based CDS interventions.

We are currently undertaking a qualitative evidence synthesis of perceptions and experiences on using CDS to implement recommendations. We will apply the GRADE-CERQual system to assess the confidence that we can have in these findings [97].

### How CDS implementers can use the GUIDES checklist

We think that the GUIDES checklist is best used by multidisciplinary teams. Each GUIDES domain is important for the outcomes of a CDS intervention, and we recommend considering all of them. For each factor, users can indicate the degree to which they think the issue has been addressed. When concerns are identified that could negatively affect the success of a CDS intervention, we recommend that the multidisciplinary group discusses the importance of these concerns and reaches a consensus about what follow-up actions are required. Further instructions are provided in the actual checklist (Additional file 1).

### How researchers can use the GUIDES checklist

The checklist may be useful for researchers seeking to identify, examine and synthesise factors potentially affecting the success of CDS. Our pilot testing with the focus group showed that the checklist could help to identify and classify important factors shaping the success of CDS interventions.

Appraisals of CDS interventions are difficult when reporting is poor. We perceive this as an important reason for the low interrater reliability that we obtained during the pilot testing. Fewer than 30% of the trials, we included provided sufficient details about the contexts in which the CDS took place or the strategy used to implement the CDS. Approximately 75% of the trials described the CDS system in sufficient detail, but half did not offer sufficient information about the advice provided by the CDS system. Without such detail, it was difficult to assess the applicability and transferability of the CDS to other settings or to identify the factors that contributed to the success or failure of the CDS interventions [98].

The STARE-HI reporting standards are a resource for improving the reporting of evaluation studies in health informatics [99]. These reporting standards recommend the inclusion of a description of the study context and details about the informatics system used. We would advise to include additional criteria in the STARE-HI standards or to develop specific reporting criteria for CDS interventions. The GUIDES checklist may be useful when considering desirable reporting criteria.

## Conclusions

We designed the GUIDES checklist to support professionals in considering, in a more structured way, the factors that may affect the success of CDS interventions. In addition to CDS implementation teams, the checklist might also support CDS developers, researchers, funders and educators. Existing frameworks, evidence and expert input informed the development of the checklist. We believe that CDS implementers who use the GUIDES checklist will get a deeper and more accurate understanding of the factors shaping CDS effectiveness and they will less likely overlook important factors.

## Additional files

Additional file 1: Expert panel feedback. (DOCX 73 kb)

Additional file 2: Patient and health consumers feedback. (DOCX 33 kb)

Additional file 3: GUIDES checklist. (PDF 901 kb)

Additional file 4: Importance ratings of GUIDES factors. (DOCX 28 kb)

## Abbreviations

CDS: Computerised decision support
GUIDES: GUideline Implementation with Decision Support
