# Supplementary material for: The GUIDES checklist: development of a tool to improve the successful use of guideline-based computerised clinical decision support
Source: Implement Sci. 2018 Jun 25;13:86. doi: 10.1186/s13012-018-0772-3 (PMC6019508; doi:10.1186/s13012-018-0772-3)
Supplement: Supplementary file 1 — Expert panel feedback. (DOCX 73 kb) [file 13012_2018_772_MOESM1_ESM.docx]

# Additional file 1

# Expert panel feedback on GUIDES checklist v1.2

**1. Are potentially important factors missing from the checklist?**

Comprehensiveness

| **Name** | **Count** | **Percent** |
| --- | --- | --- |
| **Yes** | 5 | 11.1% |
| **Uncertain** | 11 | 24.4% |
| **No** | 29 | 64.4% |
| **N** | 45 |  |

**2. Are factors included in the checklist that should not be?**

Relevance

| **Name** | **Count** | **Percent** |
| --- | --- | --- |
| **No** | 36 | 80.0% |
| **Uncertain** | 6 | 13.3% |
| **Yes** | 3 | 6.7% |
| **N** | 45 |  |

**3. Is the checklist applicable across different settings (e.g. primary and secondary care) and different types of practices (including prevention, diagnosis and treatment for chronic and non-chronic conditions)?**

Applicability

| **Name** | **Count** | **Percent** |
| --- | --- | --- |
| **Yes** | 37 | 82.2% |
| **Uncertain** | 7 | 15.6% |
| **No** | 1 | 2.2% |
| **N** | 45 |  |

**4. Is the checklist more complicated than necessary?**

Simplicity

| **Name** | **Count** | **Percent** |
| --- | --- | --- |
| **No** | 32 | 71.1% |
| **Uncertain** | 8 | 17.8% |
| **Yes** | 5 | 11.1% |
| **N** | 45 |  |

**5. Is the checklist organised in a logical way that is easy to understand?**

Logic

| **Name** | **Count** | **Percent** |
| --- | --- | --- |
| **Yes** | 42 | 93.3% |
| **Uncertain** | 2 | 4.4% |
| **No** | 1 | 2.2% |
| **N** | 45 |  |

**6. Are the factors and domains (groups of factors) labelled and explained in a way that is easy to understand?**

Clarity

| **Name** | **Count** | **Percent** |
| --- | --- | --- |
| **Yes** | 37 | 82.2% |
| **Uncertain** | 6 | 13.3% |
| **No** | 2 | 4.4% |
| **N** | 45 |  |

**7. Would researchers be capable to use the checklist?**

Usability

| **Name** | **Count** | **Percent** |
| --- | --- | --- |
| **Yes** | 36 | 80.0% |
| **Uncertain** | 9 | 20.0% |
| **No** | 0 | 0.0% |
| **N** | 45 |  |

**8. Would people who are NOT researchers be capable to use the checklist?**

| **Name** | **Count** | **Percent** |
| --- | --- | --- |
| **Yes** | 25 | 55.6% |
| **Uncertain** | 19 | 42.2% |
| **No** | 1 | 2.2% |
| **N** | 45 |  |

**9. Is the checklist suitable (appropriate) for helping people to identify factors that should be considered when implementing guideline-based CDS?**

Suitability

| **Name** | **Count** | **Percent** |
| --- | --- | --- |
| **Yes** | 40 | 88.9% |
| **Uncertain** | 5 | 11.1% |
| **No** | 0 | 0.0% |
| **N** | 45 |  |

**10. Is the checklist likely to be useful (beneficial) to people implementing guideline-based CDS?**

Usefulness

| **Name** | **Count** | **Percent** |
| --- | --- | --- |
| **Yes** | 42 | 93.3% |
| **Uncertain** | 3 | 6.7% |
| **No** | 0 | 0.0% |
| **N** | 45 |  |

**11. Overall, is the checklist adequate to be used to identify factors that determine successful use of guideline-based CDS?**

Overall assessment

| **Name** | **Count** | **Percent** |
| --- | --- | --- |
| **Yes - Could be used as is with little or no modification** | 31 | 68.9% |
| **Partially - Needs some modification or further development** | 14 | 31.1% |
| **No - Not adequate** | 0 | 0.0% |
| **N** | 45 |  |

**12. Would you use this checklist?**

| **Name** | **Count** | **Percent** |
| --- | --- | --- |
| **Yes** | 37 | 90.2% |
| **Uncertain** | 3 | 7.4% |
| **No** | 1 | 2.4% |
| **Not applicable** | 4 | - |
| **N** | 45 |  |
