# Supplementary material for: The GUIDES checklist: development of a tool to improve the successful use of guideline-based computerised clinical decision support
Source: Implement Sci. 2018 Jun 25;13:86. doi: 10.1186/s13012-018-0772-3 (PMC6019508; doi:10.1186/s13012-018-0772-3)
Supplement: Supplementary file 2 — Patient and health consumers feedback. (DOCX 33 kb) [file 13012_2018_772_MOESM2_ESM.docx]

# Additional file 2

# Feedback from patients and health consumers on the GUIDES checklist v1.2

**1. Do you think guideline-based computerised decision support is an important quality improvement intervention?**

|  | **Count** | **Percent** |
| --- | --- | --- |
| **Strongly disagree** | 0 | 0.0% |
| **Disagree** | 0 | 0.0% |
| **Agree** | 0 | 0.0% |
| **Strongly agree** | 4 | 100.0% |
| **I don't know** | 0 | 0.0% |
| **N** | 4 |  |

**2. Do you think there is a need for healthcare providers to receive computerised decision support on treatment options?**

|  | **Count** | **Percent** |
| --- | --- | --- |
| **Strongly disagree** | 0 | 0.0% |
| **Disagree** | 0 | 0.0% |
| **Agree** | 0 | 0.0% |
| **Strongly agree** | 4 | 100.0% |
| **I don't know** | 0 | 0.0% |
| **N** | 4 |  |

**3. Do you think there is need for computerised decision support on treatment options that healthcare providers and patients can discuss on during a consultation?**

|  |  |
| --- | --- |

| **Name** | **Count** | **Percent** |
| --- | --- | --- |
| **Strongly disagree** | 0 | 0.0% |
| **Disagree** | 0 | 0.0% |
| **Agree** | 2 | 50.0% |
| **Strongly agree** | 2 | 50.0% |
| **I don't know** | 0 | 0.0% |
| **N** | 4 |  |

**4. Do you think there is need for patients to receive computerised decision support on treatment options outside of a consultation with a healthcare professional?**

|  |  |
| --- | --- |

|  | **Count** | **Percent** |
| --- | --- | --- |
| **Strongly disagree** | 0 | 0.0% |
| **Disagree** | 0 | 0.0% |
| **Agree** | 3 | 75.0% |
| **Strongly agree** | 1 | 25.0% |
| **I don't know** | 0 | 0.0% |
| **N** | 4 |  |

**5. Is the GUIDES checklist a suitable (appropriate) tool to help people to identify factors that should be considered when implementing guideline-based computerised decision support?**

|  |  |
| --- | --- |

|  | **Count** | **Percent** |
| --- | --- | --- |
| **Yes** | 3 | 75.0% |
| **Uncertain** | 1 | 25.0% |
| **No** | 0 | 0.0% |
| **N** | 4 |  |

**6. Is the GUIDES checklist likely to be useful (beneficial) to people implementing guideline-based computerised decision support?**

|  | **Count** | **Percent** |
| --- | --- | --- |
| **Yes** | 4 | 100.0% |
| **Uncertain** | 0 | 0.0% |
| **No** | 0 | 0.0% |
| **N** | 4 |  |
