# Supplementary material for: The GUIDES checklist: development of a tool to improve the successful use of guideline-based computerised clinical decision support
Source: Implement Sci. 2018 Jun 25;13:86. doi: 10.1186/s13012-018-0772-3 (PMC6019508; doi:10.1186/s13012-018-0772-3)
Supplement: Supplementary file 3 — GUIDES checklist. (PDF 901 kb) [file 13012_2018_772_MOESM3_ESM.pdf]

Visit our website
www.guidesproject.org

A tool to assist professionals when implementing guidelines with computerised decision support

GUIDES CHECKLIST

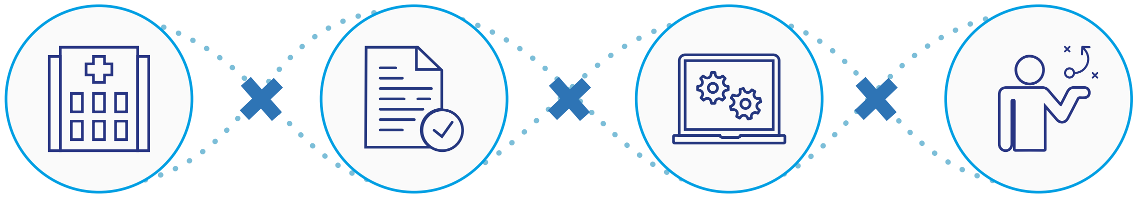

Norwegian Institute of Public Health

COLOPHON

Institution: The Norwegian Institute of Public Health, Norway
Project leader: Stijn Van de Velde
Contact: info@guidesproject.org

Norwegian Institute of Public Health

Feedback

Please send your feedback and suggestions to us at info@guidesproject.org. Please include the subject heading 'Feedback on GUIDES tool'.

Acknowledgement

We acknowledge the importance of existing frameworks and how-to books on the development of this GUIDES project. We have built on these resources to develop a detailed, comprehensive and rigorous overview of the factors affecting computerised decision support initiatives. We would like to thank the GUIDES Expert Panel for their expertise in this project, and the patients and public participants who shared their views and experiences with us. The Norwegian Institute of Public Health assumes final responsibility for the content of this publication.

Citation

Van de Velde S, Aertgeerts B, Kortteisto T, Kunnamo I, Roshanov P, Vandvik PO, Flottorp S, GUIDES Panel. GUIDES checklist - A tool to assist professionals when implementing guidelines with computerised decision support. Oslo: Norwegian Institute of Public Health, 2017.

The Research Council of Norway

UiO University of Oslo

Funding

This project received funding from the EU's Horizon 2020 Research and Innovation Programme under the Marie Sklodowska-Curie Grant Agreement No 654981. The Norwegian Research Council and the University of Oslo, Norway, provided further funding for the development of the project.

KEY MESSAGES

Computerised decision support (CDS) is a technology that provides patient-specific medical knowledge at the point of need.

CDS can improve the implementation of evidence-based recommendations, and is regarded as an important quality improvement intervention. Although the implementation of CDS is growing worldwide, the benefits remain modest. Significant investments have not consistently resulted in value for money.

The aim of the Guideline Implementation with Decision Support (GUIDES) project is to improve the use of CDS. To achieve this, we have built a checklist of factors that are likely to influence whether guideline-based CDS is successful. This checklist builds on existing frameworks, scientific evidence and expert input.

The checklist states that the successful implementation of guidelines with CDS requires:

- an enabling context
- appropriate CDS content
- an effective system
- effective CDS implementation

These four domains depend on 16 factors in total, each of which is described in the checklist.

We believe that the GUIDES checklist is an important tool for helping professionals to implement guideline-based CDS.

The GUIDES checklist is available in different formats, including an electronic version that enables CDS implementation teams to complete the GUIDES checklist efficiently in a group.

Title: GUIDES checklist

Who is this checklist for: CDS implementation teams, CDS developers, researchers, funders, educators.

Main objective: To support professionals to reflect over guideline-based CDS success factors in a structured way.

This checklist includes: Factors that can affect the success of CDS applied to implement evidence-based recommendations.

How this checklist was developed: Review and synthesis of factors affecting succesful use of CDS, collaboration with expert panel, patient and public involvement, pilot testing.

Publisher: Norwegian Institute of Public Health

Publication year: 2017

Website: www.guidesproject.org

Funding: This project received funding from the EU's Horizon 2020 Research and Innovation Programme under the Marie Sklodowska-Curie Grant Agreement No 654981. The Norwegian Research Council and the University of Oslo, Norway, provided funding for the further development of the project.

Consortium: This checklist is the product of an international collaboration between the Norwegian Institute of Public Health, Katholieke Universiteit Leuven, MAGIC, Duodecim, and McMaster University.

short overview of the

GUIDES CHECKLIST

The GUIDES checklist provides an overview of success factors for guideline-based CDS and supports professionals to reflect over these factors in a structured way.

The website www.guidesproject.org provides access to an electronic version that enables CDS implementation teams to complete the GUIDES checklist efficiently in a group.

Domain 1: CDS context

1.1 CDS can achieve the defined quality objectives

1.2 The quality of the patient data is adequate

1.3 Stakeholders and users accept CDS

1.4 CDS can be added to the existing workload, workflows and systems

Domain 2: CDS content

2.1 The content provides trustworthy evidence-based information

2.2 The decision support is relevant and accurate

2.3 The decision support provides an appropriate call to action

2.4 The amount of decision support is manageable for the target user

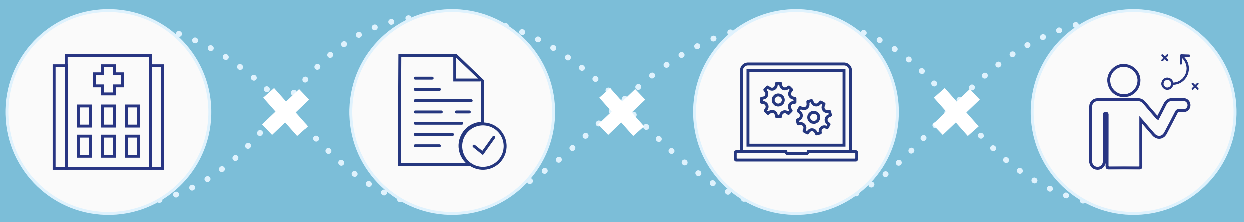

4 Domains that impact on the success of CDS to implement recommendations.

Domain 3: CDS system

3.1 The system is easy to use

3.2 The decision support is well delivered

3.3 The system delivers the decision support to the right target person

3.4 The decision support is available at the right time

Domain 4: CDS implementation

4.1 Information to users about the CDS system and its functions is appropriate

4.2 Other barriers and facilitators to compliance with the decision support advice are assessed/addressed

4.3 Implementation is stepwise and the improvements in the CDS system are continuous

4.4 Governance of the CDS implementation is appropriate

This project was headed by the Norwegian Institute of Public Health and has received funding from the EU's Horizon 2020 research and innovation programme.

Norwegian Institute of Public Health

WHO WE ARE

The following individuals contributed to the preparation of the GUIDES checklist:

Project leader

Stijn Van de Velde

Project group

Bert Aertgeerts, Signe Flottorp, Tiina Kortteisto, Ilkka Kunnamo, Pavel Roshanov, Per Olav Vandvik

GUIDES panel

Experts: Smisha Agarwal, Leila Ahmadian, David Bates, Linn Brandt, Romina Brignardello-Petersen, Carl Cauwenbergh, Yaolong Chen, Nicholas Conway, Nicolas Delvaux, Pierre Durieux, Robert El-Kareh, Atle Fretheim, Robert Greenes, Robert Brian Haynes, Annemie Heselmans, Tim Holt, Robert A. Jenders, Kensaku Kawamoto, Tamara Kredo, Edwin Lomotan, Marjolein Lugtenberg, Luis Marco-Ruiz, Colin McCowan, Lisa McDermott, Stephanie Medlock, Maria Michaels, Blackford Middleton, Marc Mitchell, Lorenzo Moja, Michael Mugisha, Jerome A. Osheroff, Pablo Alonso Coello, Sallie-Anne Pearson, Sylvia Pelayo, Joshua Richardson, Peeter Ross, Nard Schreurs, Matthew Semler, Dean Sittig, Tigest Tamrat, Madis Tiik, Anna Turusheva, Heleen van der Sijs, Robert Vander Stichele, Mieke Vermandere, Abigail Viall, Mahima Venkateswaran, Adam Wright, Taryn Young

Public and patient participants: Tom Codron, Kari Håvelsrud, Sarah Rosenbaum, Satu Salonen

Consortium

This checklist is the product of an international collaboration between the following organisations

INTRODUCTION

Background

In health systems, patient care must be based on the highest-quality relevant medical knowledge. Evidence-based guidelines help healthcare providers and patients to make well-informed healthcare decisions[1]. Unfortunately, there are gaps in the journey from the publication of evidence to the implementation of that evidence in clinical practice.[2, 3] This may cause excessive preventable mortality and morbidity and impacts on healthcare costs.

Changes in healthcare practice are not achieved simply by publishing guidelines.[4] Specific implementation strategies are also needed, such as the use of computerised decision support (CDS). This technology uses patient-specific data (for example a diagnosis, a prescription, or a combination of data elements) to provide relevant medical knowledge at the point-of-need. Functions include: offering healthcare providers relevant recommendations, informing them about care tasks based on guideline recommendations, providing patient-directed advice to empower and motivate patients, and facilitating shared decision making by presenting links to decision aids.

Rationale for the GUIDES checklist

Substantial investments have been made in healthcare information technology with decision support capabilities. However, despite popular claims, the benefits of CDS remain modest.[5, 6] Many initiatives have fallen short of expectations and,[7] in some instances, unintended consequences have led to patient harm.[8, 9]

CDS is a complex intervention and its success (or failure) is affected by many factors. The implementation of CDS systems is growing, and is being applied to increasingly larger and more complex interventions. The first published trials of CDS date back to the 1970s. However, we are still developing an understanding of which factors, or combinations of factors, make CDS effective.[5, 10] Caution is therefore needed to ensure that CDS is applied in the best possible way.[11]

Given the complexity of CDS, we argue that a structured approach to implementation is important for professionals because it facilitates a deeper and more accurate understanding of which factors make CDS more (or less) effective . Relying on less structured, individual approaches can lead to key factors and influences being overlooked. The approach, we suggest, is the use of a checklist to guide CDS implementation.[12]

Objectives of the GUIDES checklist

The purpose of the GUIDES checklist is to help professionals to reflect, in a more structured way, on the factors affecting the success of CDS interventions. The overall aim of the checklist is to increase the success of guideline-based CDS in terms how they improve healthcare, health outcomes, cost management, and patient and provider satisfaction.[13]

This checklist is designed to be flexible and applicable to many settings and uses, including different:

- Contexts such as high-, middle- and low-income countries.
- Healthcare settings such as primary, secondary and tertiary care and such as small and large practices.
- Types of care practices including recommendations related to prevention, diagnostic tests and strategies, and treatment and follow-up for chronic and non-chronic conditions.
- Targeted uses such as healthcare provider-directed information, patient-directed information or both.
- Types of CDS functions such as data presentation, alerts, reminders, references to supporting information, computerised order entry systems, dose calculators, medication reviews, calculations of prediction rules and severity-of-illness assessments, shared decision-making tools, and population-based functions.

Every CDS tool and setting has unique characteristics and we recognise that some elements in this checklist are not applicable to every context.

> While the focus of this work is on CDS, we recognise that CDS is not a magic bullet. Particular circumstances may require other implementation interventions or multifaceted strategies.[14] Any decision to use CDS or other, additional interventions should be based on an assessment of the determinants of healthcare practice that affect whether the desired changes can be achieved.[15]

Scope of the GUIDES checklist

The scope of the checklist includes:

- Guidelines: because these are systematically developed statements to inform healthcare provider and patient decisions about appropriate healthcare for specific clinical circumstances.[1] CDS can implement both strong and weak recommendations* and these can be either in favour of or against a particular medical strategy.[16, 17] Other types of evidence can also provide valuable CDS content and are also included in the scope.
- Computer-based decision support: because the use of a computer, rather than a manual process, to generate decision support has been identified as a more effective approach.[18] Manually generated decision support may provide a relatively cheap alternative to CDS, provided it is effective.[19]

What the GUIDES checklist is not:

- The checklist is not a tool for evaluating the quality of guidelines or evaluating the implementability of a guideline recommendation into CDS. Please refer to the AGREE II instrument and the GLIA instrument for more information on how these can be assessed.[20, 21]
- The checklist is not a tool for choosing CDS targets. For that purpose, please consult "Improving Outcomes with Clinical Decision Support: An Implementer's Guide".[22]
- The checklist does not address the succesful use of CDS expert systems for proposing a diagnosis, conducting a risk assessment, or predicting a prognosis without accompanying guideline-based advice.

---

* Strong recommendations are situations where almost all informed people would make the recommended choice and weak recommendations are situations where most well informed people would make the recommended choice, but a substantial minority would not. When a recommendation is strong, a decision typically takes less time and the focus of the implementation will usually be on increasing guideline adherence. A weak recommendation, in contrast, implies that there is greater variability in choices. More attention and time needs to be spent on the process of shared decision making when working with weak recommendations.[16]

USERS GUIDE

Target users

The primary intended users of this GUIDES checklist are CDS implementation teams that are planning a guideline-based CDS strategy. In addition, the checklist might also support other users with diverse interests, including:

- CDS system developers who want to evaluate their own products and find ways to optimise their CDS solutions according to key system and content factors.
- Guideline developers assessing the potential of CDS as an implementation strategy.
- Researchers seeking to identify, examine and synthesise the factors potentially affecting the success of CDS efforts.
- Funders deciding which CDS solutions to acquire and how to implement them.
- Educators helping to teach key factors for guideline-based CDS.

This checklist is designed for different users, ranging from those with only a basic knowledge of CDS, to people who are experts in CDS. We recommend that experts are always involved when applying the GUIDES checklist.

Summary of GUIDES checklist content

The checklist contains four CDS domains:

1. The CDS context domain focuses on the circumstances in which CDS can be potentially successful;
2. The CDS content domain focuses on the factors shaping the success of the advice produced by the CDS system;
3. The CDS system domain focuses on the features belonging to the CDS tool;
4. The CDS implementation domain refers to the factors affecting the CDS integration in practice settings.

It is important that all of these domains are considered when trying to achieve or sustain intended CDS outcomes. The successful implementation of evidence-based CDS recommendations can be expressed in the diagram shown below. This diagram is adapted from the formula by Fixsen on successful uses of evidence-based programs in human service settings.[23]

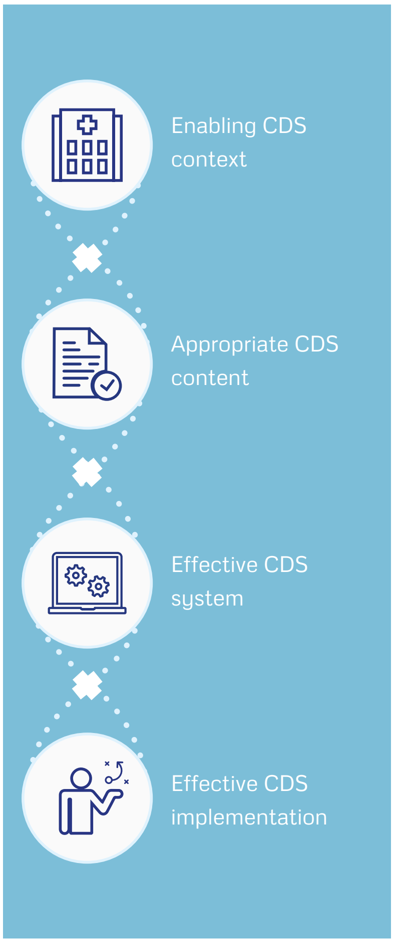

Each domain includes four factors, and there are 16 factors in total:

- All the factors listed are important and all must be considered. The level of importance of the various factors may vary in specific circumstances (for example, "Governance of the CDS implementation is appropriate" may be less important in a small practice setting).
- In most instances, it is clear why each factor has been assigned to a particular domain. We recognise that in a few instances factors could be assigned to more than one domain (e.g. the factor "The system delivers the decision support directly to the right target person" has a relation to both the CDS system domain and the implementation domain). Some factors are also interrelated, such as "Stakeholders and users accept CDS", which is also affected by factors in the content, system and implementation domain.
- We provide a rationale explaining the importance of each factor, present sample questions to consider, and include an answer scale. The printed and online versions include space for notes.
- The rationale can either be an assumption or it can be supported by scientific evidence. Statements on the level of confidence that a factor affects the outcomes of a CDS initiative will be published in a separate systematic review report.[24, 25]

The GUIDES checklist is available in four different formats:

- An overview diagram provides a meta-view of the 4 domains and the 16 factors (see page 4).
- A full checklist describes each factor in

detail (see pages 11-28).

- A short version of the checklist includes a list of the 16 factors, as well as 'how to evaluate CDS' questions (see Annex 1).
- The electronic version of the checklist presents the GUIDES checklist in a layered format and enables CDS implementation teams to complete the GUIDES checklist efficiently online in a group (available on www.guidesproject.org).

Practical issues when using the GUIDES checklist

- We suggest that those wishing to use the GUIDES checklist should create a multidisciplinary team in which different specialists engage in multidisciplinary discussions. Participants could include healthcare providers, experts in developing and applying CDS, health service managers, guideline developers and researchers. Involving patients can help multidisciplinary groups to obtain a wider range of views and can help to ensure that the adopted strategies are relevant and acceptable to patients.[26] Patients can be consulted by the multidisciplinary group or can participate directly in the group.
- The GUIDES checklist is not simply a box-ticking exercise. Users must make their own judgements about how particular factors affect CDS initiatives. Clear communication about the purpose of the GUIDES checklist is required, and a commitment from all participants that they will use the checklist.
- Each GUIDES domain is important for the outcomes of a CDS intervention and we recommend considering all of them. The most logical approach is to consider the context domain first because it provides a way to double-check that CDS is an appropriate implementation strategy. It is possible to focus on individual domains at different stages of the intervention development process.
- The 'how to evaluate' questions listed for each factor, are designed to help users reflect on all those components that are important for the assessment of a given factor. For each factor, users can indicate the degree to which they think the issue has been dealt with. The categories range from 'strongly disagree' to 'strongly agree'. The notes section allows users to add information relevant to how they made a judgement and to include information about any of the follow-ups required.
- Users may find that not all the considerations for a given factor are met, in which case, they should give a 'disagree' response. Such a 'disagree' response may indicate that there are concerns that could negatively affect the results of a CDS intervention. In such instances, we recommend that the multidisciplinary group discusses the importance of these concerns, and reaches a consensus about what follow-up actions are required.

CDS interventions can either be focused and relate to only a small set of recommendations, or they may be comprehensive and relate to many topics. Comprehensive CDS approaches, we suggest, should evolve gradually to full-scale implementation. Those responsible for the implementation of comprehensive CDS approaches may choose to focus on only a particular number of CDS targets at a time. In the case of comprehensive CDS scenarios, it might be possible to examine some checklist factors in batches for multiple CDS targets simultaneously; while other factors may need to be addressed individually for each target.

If CDS is part of a multifaceted quality improvement strategy, then careful reflection is also needed on how to implement the co-interventions in the most effective way. The GUIDES checklist does not provide support on how to design other interventions than CDS.

HOW THE GUIDES TOOLS WERE DEVELOPED

To ensure the relevance, quality and usefulnessof the GUIDES tool, the following tasks were completed during its development[27]

1

REVIEW

We reviewed the research evidence and frameworks on the determinants of implementing recommendations using CDS.

2

SYNTHESIS

We completed a synthesis of a comprehensive checklist for the determinants identified.

3

EXPERT FEEDBACK

We obtained feedback and input about the tool by collaborating with an international group of experts and by collecting views from patient and public representatives.

4

PILOT TESTING

We pilot-tested the tool by using it to conceptualise a tailored CDS intervention and by testing it in a systematic review of trials on CDS.

FURTHER DEVELOPMENT

We will continue to systematically apply the checklist in CDS efforts. This will help to improve the checklist. We hope to evaluate our own experiences and the experiences of other projects in the diverse settings in which the GUIDES checklist is used. Feedback about the GUIDES checklist can be provided by sending an email with the subject heading ‘Feedback on GUIDES tool’ to info@guidesproject.org.

THE GUIDES CHECKLIST

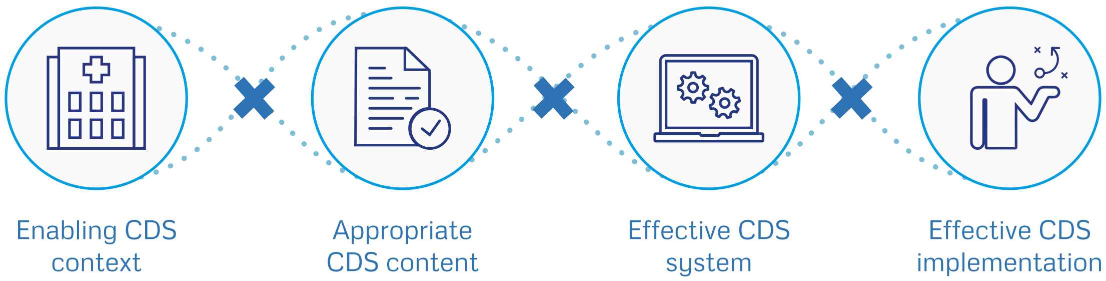

DOMAIN 1: THE CDS CONTEXT

1.1 CDS can achieve the defined quality objectives

☐ Strongly disagree ☐ Disagree ☐ Somewhat disagree ☐ Undecided ☐ Somewhat agree ☐ Agree ☐ Strongly agree

Notes:	Follow-up actions:

Rationale

A CDS intervention must attempt to address the factors that explain the current behaviour of healthcare providers and patients regarding the desired quality objectives. CDS is just one of many possible quality improvement strategies. Depending on the factors that affect the behaviour of healthcare professionals and patients, other strategies might be more appropriate, or additional interventions may be required to achieve the desired outcomes (see 4.2).

The effectiveness of CDS may vary by outcome types (patient, process or system outcomes), the types of tasks envisaged (e.g. prevention, diagnosis, treatment, prescribing, test ordering, imaging, health-related behaviour changes), and by the settings in which CDS is implemented (e.g. in an outpatient, inpatient, emergency department, or intensive care unit setting).

References: [22, 28-52]

How to evaluate

Consider the following questions:

- Does CDS address the factors that explain the current behaviour of healthcare providers and patients?
- Does the available evidence support the use of CDS for the given outcomes, tasks and settings?

Examples

Positive examples could include:

- Few patients are currently receiving an appropriate form of care. The CDS intervention being considered has been used successfully elsewhere. It is likely that the results will be transferable.
- A lack of knowledge about the recommended practice for a targeted problem is an important determinant shaping the current suboptimal quality of care. It is likely that CDS can address this determinant.
- Based on an analysis of the determinants of the care currently provided, a multifaceted quality improvement strategy has been planned. CDS is one of the included interventions.

Negative examples could include:

- Baseline performance levels are already high. Introducing CDS may therefore probably have little or no benefit.
- Available evidence showing high incidences of CDS being ignored or overridden for similar objectives in other settings is not taken into account.
- The suboptimal quality of care is primarily explained by organisational regulations or a lack of resources. These issues cannot be addressed by CDS.

1.2 The quality of the patient data is adequate

☐ Strongly disagree ☐ Disagree ☐ Somewhat disagree ☐ Undecided ☐ Somewhat agree ☐ Agree ☐ Strongly agree

Notes:	Follow-up actions:

Rationale

The effectiveness of CDS depends on the availability of high-quality patient data. Inaccurate or incomplete patient data may lead to false positive decision support (i.e. irrelevant advice) or false negative decision support (i.e. no advice for patients who are at risk). Some CDS systems compensate for data deficiencies or can be used to record complete and up-to-date patient data.

Data quality depends on the type of data systems used to capture the data (e.g. electronic health records) and on their appropriate use. Data standards (such as HL7 vMR, HL7 FHIR, openEHR, and ISO13606) help to ensure data quality by defining the structure of how the clinical information is recorded in data fields.

A CDS system must be able to interpret patient data. A prerequisite for doing so is the use of terminologies (such as SNOMED-CT, LOINC, ICPC2, and ICD-10) that assign meaning to the content of data fields. The use of appropriate data standards and terminologies enable semantic interoperability and allow information to be shared across health information systems. They also allow the same conclusions to be derived from the same data sets if the same inference methods are applied in different contexts.

References: [22, 28-31, 33-35, 37, 39, 44, 51, 53-61]

How to evaluate

Consider the following questions:

- Is the structured patient data that is needed to achieve the CDS objective sufficiently accurate and complete to allow the use of CDS?
- If necessary, can the quality of the data be improved or can the CDS itself improve the data quality?

Examples

Positive examples could include:

- Prior to the intervention, quality assurance staff and healthcare providers evaluate and optimise the quality of the patient data in the patient records.
- In the waiting room, patients fill in a form which is transmitted to the healthcare provider. The system provides advice based on this data.
- The system prompts users to verify data that may no longer be valid (e.g. their pregnancy status).
- The presence of a medical condition is determined by analysing diagnosis codes, drugs, and laboratory tests rather than by analysing only the diagnosis codes.

Negative examples could include:

- Gaps in the treatment history of patients leads to cases of erroneous decision support. This erodes confidence in the system and results in users ignoring reminders or stopping their use of the CDS system.
- Healthcare providers do not record certain patient problems or interventions because the data standards do not describe particular diagnoses adequately.
- Suboptimal data quality leads to incorrect advice that makes some healthcare professionals change their initially correct treatment decisions.

1.3 Stakeholders and users accept CDS

- [ ] Strongly disagree
- [ ] Disagree
- [ ] Somewhat disagree
- [ ] Undecided
- [ ] Somewhat agree
- [ ] Agree
- [ ] Strongly agree

Notes:

Follow-up actions:

Rationale

Acceptability and acceptance are multifactorial concepts. In the context of CDS, these can be shaped by how a CDS system works, the visual display used, the advice provided, how CDS is implemented, and by individual and group attitudes towards CDS. Here we focus on the attitudes toward the CDS intervention.

A CDS intervention is more likely to be used if people see a direct benefit or expect that it will have a positive impact on a major health problem, or have outcomes that are of importance to patients.

The acceptance of a CDS system can be hindered if users find it annoying, patronising or threatening. External rules, regulations and pressures may also influence attitudes towards implementing and using CDS.

It is important that all stakeholders (including health service managers, funders, patients, healthcare providers) are consulted to ensure that the CDS strategy chosen is relevant and acceptable.

References: [22, 32, 34, 36, 43-45, 47, 51, 52, 57, 58, 61-76]

How to evaluate

Consider the following questions:

- Is there a clear benefit to the users who will engage with the CDS?
- Do the users and stakeholders have a positive attitude towards the use of CDS?
- If necessary, is it possible to increase user and stakeholder acceptance?

Examples

Positive examples could include:

- The CDS and the guidelines on which it is based have broad support from healthcare providers within the institution applying the CDS.
- The healthcare providers expect the decision support to have a positive impact on the patient care.
- Local opinion leaders are successfully advocating the priority introduction of CDS.

Negative examples could include:

- The healthcare providers perceive the CDS system to be a threat to their communication with patients or as a threat to their clinical autonomy, medical liability, or their professional privacy.
- The healthcare providers believe that CDS support is, in effect, a formulaic 'cookbook' approach to medicine and that the primary purpose of the advice is to decrease healthcare costs.
- The healthcare providers distrust the CDS system because they think it may fail to protect patient privacy and patient data.
- The introduction of CDS may not be regarded as a priority by healthcare providers because of other ongoing quality improvement projects.

1.4 CDS can be added to the existing workload, workflows and systems

☐ Strongly disagree ☐ Disagree ☐ Somewhat disagree ☐ Undecided ☐ Somewhat agree ☐ Agree ☐ Strongly agree

Notes:	Follow-up actions:

Rationale

Installing and using new CDS may cause technical difficulties and negatively impact the existing information systems and CDS solutions already in place.

Healthcare providers typically have large workloads and time to read and act upon the decision support can be an issue. Success is more likely if the CDS fits well within existing routines and workflow processes. It can be difficult to change existing procedures or responsibilities and alterations may lead to unintended negative consequences.

CDS has the potential to improve workloads and work processes, but it can also create workflow inflexibility and workarounds may burden healthcare providers or negatively affect patient care.

References: [28-30, 32, 36, 37, 39, 43, 44, 51, 52, 57-61, 63, 65-67, 74, 77, 78]

How to evaluate

Consider the following questions:

- Is the required hardware available and what will the impact be of adding CDS to the existing information systems?
- Is it feasible to introduce CDS, given the current workload and the usual work processes?
- If necessary, can the workload or the work processes be changed or can the CDS system improve the workload or work processes?

Examples

Positive examples could include:

- Every exam room has a computer which supports CDS; computers are also available in common work areas.
- The workflow of the healthcare providers is carefully studied before the introduction of the system and the system is customised to fit in with the current workflow.
- The scope of the CDS intervention is limited to respect the time constraints of the healthcare providers.
- The CDS is designed to streamline and automate the workflow.

Negative examples could include:

- There is no infrastructure to support the use of CDS at bedsides during ward rounds or during patient home visits.
- Healthcare providers work in pressured practice settings and lack the time to use the CDS system
- Currently, healthcare providers are only inputting data or using electronic health records at the end of their interactions with patients. It is unlikely that this routine can be changed easily and this therefore limits the potential effectiveness of the CDS effort.
- The introduction of CDS eliminates the current verification of treatment orders by other health personnel. Shifting this human role to a computer role may have unintended negative consequences.

DOMAIN 2: THE CDS CONTENT

2.1 The content provides trustworthy evidence-based information

☐ Strongly disagree ☐ Disagree ☐ Somewhat disagree ☐ Undecided ☐ Somewhat agree ☐ Agree ☐ Strongly agree

Notes:	Follow-up actions:

Rationale

CDS-guided decisions about diagnosis, prevention, treatment and follow-up must be based on the best current evidence available, typically from clinical practice guidelines that meet standards of trustworthiness.[79] It is important that CDS decision support is clear about the benefits and harms of the management options available, the certainty of the evidence, the importance of the outcomes for patients, and the acceptability and feasibility of the intervention.[79, 80]

Providing such information can help healthcare providers and patients to make better-informed healthcare decisions, and helps them to critically appraise the decision support.

Users must be able to critically appraise the recommendations by, for example, checking the underlying original research. To accomplish this, users should be able to move easily from the CDS advice to the findings of related systematic reviews and primary studies.

A credible and transparent knowledge management plan is needed to ensure that the decision support content can be kept up-to-date after the introduction of the CDS system (see also 4.4).

References: [22, 29, 31-34, 36-39, 44, 47, 49-51, 55, 57-59, 62, 66, 68, 71, 77, 81-84]

How to evaluate

Consider the following questions:

- Do the organisation(s) and people that developed the decision support have credibility?
- Is the advice supported by up-to-date scientific evidence and is the type and quality of this evidence clear to the user?
- Is the decision support clear on the benefits and harms of the different management options?

Examples

Positive examples could include:

- An expert panel is developing the decision support using trustworthy guidelines and a comprehensive review of the available evidence. Formal methods are helping the panel to reach consensus.
- The decision support is backed up by detailed recommendations that clearly communicate the strength of the recommendations and the balance between the desirable and undesirable effects of adherence to the management options.
- The methods to develop and update the decision support are explicitly described and users can find this information easily.

Negative examples could include:

- The evidence used in the decision support content is of low quality.
- The decision support provides practical background information about the advice but there is no link to the supporting recommendations.
- New research contradicts the existing decision support and there is no capacity to update the advice.

2.2 The decision support is relevant and accurate

☐ Strongly disagree ☐ Disagree ☐ Somewhat disagree ☐ Undecided ☐ Somewhat agree ☐ Agree ☐ Strongly agree

Notes:	Follow-up actions:

Rationale

Decision support must be relevant to the information needs of the users. If not, it can be a burden to them.

Inaccurate decision support may cause distrust among users or lead to inappropriate decisions. The degree of accuracy depends on the quality of the patient data (see 1.2) and on the sophistication of the decision support system to respond to the patient data.

Systems that explain why the decision support was triggered may help to establish trust in the CDS system. They may also enable users to judge if the decision support is relevant and accurate.

References: [22, 29, 31-34, 36, 38, 44, 49-51, 54, 55, 57-60, 62, 63, 65, 66, 73, 74, 81, 83-86]

How to evaluate

Consider the following questions:

- Does the decision support contain accurate information that is pertinent to the care of the patient?
- Does the decision support address the information needs of the users?
- Is it clear to the users why the decision support information is provided for a given patient?

Examples

Positive examples could include:

- The system has been pilot tested and the healthcare providers involved agree that the decision support advice is relevant and accurate.
- The decision support comes with an explanation about why the decision support was triggered.
- Some recommendations are well adhered to by experienced practitioners, but less adhered to by inexperienced providers. The system makes it possible to customise who receives which decision support.

Negative examples could include:

- The system provides advice for situations in which healthcare providers would also choose the recommended action without receiving any decision support.
- The system does not take co-morbidities into account and this may lead to inaccurate decision support.
- The decision support advice does not take varying baseline risks into account and is not clinically important for every patient.
- The CDS for the targeted objective is unlikely to be beneficial because of the complexity of the patient cases

2.3 The decision support provides an appropriate call to action

☐ Strongly disagree ☐ Disagree ☐ Somewhat disagree ☐ Undecided ☐ Somewhat agree ☐ Agree ☐ Strongly agree

Notes:	Follow-up actions:

Rationale

Decision support systems should be clear about the actions required and on the clinical importance and urgency of the actions. The information must be presented in ways that are easy to understand and process. Clearly written information is especially important when advice is directed at patients.

Changing a behaviour by recommending an action or other choice may be easier than simply trying to stop an intended action. However, suggesting alternatives might be difficult if the advice depends on other factors that CDS cannot easily take into account. Alternative options should be presented with trustworthy information about the possible benefits and harms of adhering to them.

An appropriate call-to-action also implies that the decision support is directly applicable in the setting where the advice is generated. If not, adherence to the decision support will be hindered.

References: [29-31, 33, 34, 38, 39, 41, 44, 55, 60, 62, 82, 83]

How to evaluate

Consider the following questions:

- Is the clinical importance and urgency of the recommended action sufficiently clear?
- Is the recommended action clear enough for the targeted users to act on?
- Is the advice applicable in the setting in which it will be implemented?
- Is it clear how the recommended action fits with other current guidelines?

Examples

Positive examples could include:

- The readability of the CDS is tested and adaptations are made based on the findings.
- The CDS recommends not doing an intended action and lists alternative treatment options and links to more information these alternatives.
- The CDS evaluates the patients baseline risks and provides an individualised estimation of the balance of benefits and harms of the potential interventions.
- A CDS for services with waiting lists estimates the earliest possible dates that users will be able to access the recommended services.

Negative examples could include:

- Some of the recommended clinical interventions are not available in the setting in which the system is implemented.
- The decision support advice contradicts local customs or norms, or other current guidelines.
- The generated decision support displays patient data (e.g. lab values) that is outside normal values, but it is not clear to the users how they should act upon it.

2.4 The amount of decision support is manageable for the target user

☐ Strongly disagree ☐ Disagree ☐ Somewhat disagree ☐ Undecided ☐ Somewhat agree ☐ Agree ☐ Strongly agree

Notes:	Follow-up actions:

Rationale

Ranking and displaying issues in order of importance helps to make decision support easier and allows users to focus on the most important issues first.

Providing too much decision support recommendations could burden healthcare providers and reduce their commitment and interest. If CDS leads to a large amount of decision support, it might be necessary to implement a co-intervention (e.g. a staff-oriented strategy) that allows the healthcare providers to process the recommendations within the time constraints.

References: [22, 30, 31, 33, 34, 51, 54, 55, 57, 59, 60, 62, 66, 73, 74, 84, 85, 87]

How to evaluate

Consider the following questions:

- Is the amount of decision support per patient manageable?
- Is the total amount of decision support manageable for the healthcare provider?

Examples

Positive examples could include:

- The decision support provides an indication of the clinical importance of a decision and ranks it accordingly.
- The threshold for displaying decision support can be changed according to variations in the priority levels of the issues that require attention.
- A large amount of drug-related decision support leads to a full medication review.

Negative examples could include:

- The amount of suggested treatment options is overwhelming and the patient and the healthcare professional find it hard to make a choice.
- The healthcare providers find that the intensity of the decision support is too high. Consequently, they ignore the advice or deactivate the system.
- The number of recommendations generated by the decision support is too high and slows down the provision of healthcare.

DOMAIN 3: THE CDS SYSTEM

3.1 The system is easy to use

☐ Strongly disagree ☐ Disagree ☐ Somewhat disagree ☐ Undecided ☐ Somewhat agree ☐ Agree ☐ Strongly agree

Notes:	Follow-up actions:

Rationale

If a system is slow or complex to use, or if it crashes frequently, users may become frustrated, use it less, or stop using it completely. International standards are available to evaluate the usability of software in general.[88, 89]

To stimulate healthcare providers to use a CDS system, the time needed to add or correct patient data should be minimal.

Adherence is more likely if a system helps users to complete the recommended actions.

Healthcare providers prefer systems that interface easily with other computerised information systems. Better integration helps to prevent systems from becoming fragmented and having to change applications.

User needs and preferences may vary and customisable systems may therefore be more useful.

References: [22, 28-31, 33, 34, 36, 37, 43, 44, 47, 54, 55, 58-63, 65-68, 71, 74, 76, 82-84, 90, 91]

How to evaluate

Consider the following questions:

- Is it easy for users to interact with the CDS system?
- Does the system facilitate (or, at least, not hinder) the workflow of the healthcare providers?
- Can the system be customised to provide better user support
- Is the system always up and running?

Examples

Positive examples could include:

- The IT hardware can provide suitable, stable and fast CDS.
- After pilot testing a system, the healthcare providers found that its impact on clinical work was acceptable.
- The system interacts with other computerised information systems and healthcare providers can do most of their tasks within the same application.
- The CDS prepopulates a treatment order with recommended actions (drugs, tests, or procedures)

Negative examples could include:

- Patient data entered using the CDS is not automatically stored within the electronic health record of a patient and information must be entered twice.
- The system requires a lot of user effort (e.g. too many mouse clicks, scrolling, window changes, password prompts, etc.
- Limited interfaces between the decision support system and the order entry system creates a significant hurdle.
- Regularly CDS system updates create too much downtime.

3.2 The decision support is well delivered

☐ Strongly disagree ☐ Disagree ☐ Somewhat disagree ☐ Undecided ☐ Somewhat agree ☐ Agree ☐ Strongly agree

Notes:	Follow-up actions:

Rationale

To maximise the potential efficiency and effectiveness of CDS, a suitable delivery mode (system-initiated, on-demand), format (electronic, paper) and channel (electronic record, order entry system, mobile device, etc.) should be chosen. These choices will depend on the setting in which a CDS is located and will require knowledge of users' workflows and preferences.

System-initiated advice which is automatically provided within a clinical workflow may make healthcare professionals aware of an information need or can prevent safety errors. Making information available on user request can also be relevant when it is needed at a later stage in the workflow or for decision support that has a lower priority level.

To ensure that the information provided to users is noticed and easy to process, CDS information displays should be eye-catching, intuitive, concise, and consistent. Ambiguous or confusing information may lead to errors in decision making and should be avoided.

To improve user understanding, information could be presented, for example, in a layered format in which the key information is displayed first, and additional content is provided in expandable sub-layers.[92]

CDS systems can include specific functions designed to prevent CDS advice from being overlooked or neglected. These functions may include:

Requiring practitioners to indicate (dis)agreements with the CDS advice. Practitioners may be more likely to adhere to recommendations if they have to record justifications of individual decisions that may be visible to others.

Restricting clinical actions by using CDS may be a useful approach in instances in which there are severe potential patient risks.

Intrusive decision supports (e.g. pop-ups) can help to draw users' attention to prioritised decision support.

However, such functions can also have unintended negative consequences. They may contribute to alert fatigue, they may hinder urgent responses and they may lead to unintended workarounds (e.g. practitioners bypass the documentation process)

How to evaluate

Consider the following questions:

- Is the advice delivered in an appropriate mode, format and channel?
- Is the display of the decision support eye-catching, intuitive, concise, consistent and unambiguous?
- Is it appropriate to use specific functions (e.g. pop-ups, computerised restrictions, indications of (dis)agreement) for prioritised decision support?

References: [22, 29-34, 36, 38, 39, 41-44, 47, 49, 51, 52, 55, 57, 58, 62, 63, 66, 67, 72, 74, 82-84, 93, 94]

Examples

Positive examples could include:

- In a decentralised home care setting, the decision support is provided through automatic emails.
- The decision support is provided on paper when it is not possible to access CDS during a patient interaction.
- The system uses visually clear formats (e.g. large lettering, colours) to highlight terms that may be easily confusable, such as very similar-looking drug names, or to distinguish between recent and older test results.

Negative examples could include:

- An icon indicating that decision support is available is often unnoticed among other available icons.
- Decision support is always provided as an intrusive alert and does not differentiate between priority and non-priority situations.
- The decision support advice is presented with colour codes and icons, but it is not clear for users what these mean.
- Users find it frustrating that the system requires them to state their reasons for non-compliance. Some healthcare providers choose to comply or to add untrue data simply to avoid extra work.

3.3 The system delivers the decision support to the right target person

☐ Strongly disagree ☐ Disagree ☐ Somewhat disagree ☐ Undecided ☐ Somewhat agree ☐ Agree ☐ Strongly agree

Notes:	Follow-up actions:

Rationale

Systems that provide advice directly to those who are responsible for making a clinical decision might be more successful than systems that provide advice through an intermediary. When selecting the 'right' people, it is important to have a good understanding of the factors that affect the behaviour of healthcare professionals and patients for the CDS target (see 4.2). A target person may be a health personnel, for example, a patient, or both.

Some systems can improve the quality of care by facilitating collaboration between the healthcare providers who are providing care for a specific patient.

References: [22, 29, 32-34, 37, 39, 41, 44, 46, 47, 51, 55, 62, 67, 72, 82, 84, 85, 95]

How to evaluate

Consider the following questions:

- Is the system reaching the targeted users (healthcare providers and/or patients)?
- Is the system able to facilitate team processes when these are needed?

Examples

Positive examples could include:

- Prior to the interaction with the patient, the system sends information to both the patient and the healthcare provider about current health risk factors and the management options to address these.
- The system provides access to tools for shared decision making that can be displayed and printed during the interaction with the patient.
- The decision support and the response of the targeted decision maker are visible to the other healthcare providers caring for the same patient. This helps them to improve the team processes together.

Negative examples could include:

- The information is channelled to an intermediary person and does not routinely reach the targeted decision maker.
- Directing decision support towards patients could help to overcome the indifference that some healthcare providers may have about a CDS target, but this strategy is not possible for logistical reasons.

3.4 The decision support is available at the right time

- ☐ Strongly disagree
- ☐ Disagree
- ☐ Somewhat disagree
- ☐ Undecided
- ☐ Somewhat agree
- ☐ Agree
- ☐ Strongly agree

Notes:	Follow-up actions:

Rationale

Decision support needs to be available at the point of need. This may be before, during, or after a patient interaction, or independent of it. Some CDS focuses on one decision only; other CDS systems might have to deal with a complex sequence of consecutive interventions over a period of time. The appropriate timing of the advice depends on the targeted quality problem, the clinical reasoning process involved, and on user preferences.

References: [22, 29, 31, 33, 34, 36, 38, 39, 41, 44, 47, 51, 59, 62, 67, 82-84]

How to evaluate

Consider the following question:

- Does the system provide the decision support at a moment of need?

Examples

Positive examples could include:

- The decision support is available to healthcare providers before their patient encounters and helps them to be better prepared when seeing patients.
- The system guides healthcare providers through the clinical workflow and provides advice in real-time. This allows users to consider the advice before they make any decisions.
- Time pressures experienced by healthcare providers may mean that they prefer to use the CDS system after they have completed their clinical work. The system allows the decision support to be accessed at this point instead.
- The system reminds healthcare workers about the need to conduct periodic medication reviews and supports users in identifying medication-related problems.

Negative examples could include:

- Decision support only becomes available after a treatment choice is made and the data are entered in the system.
- The decision support is provided independently of contact with the patient and may be forgotten during the contact.
- The CDS suggests stopping an action that has already occurred and the healthcare professional has to interrupt the workflow to revert the initial action.

DOMAIN 4: THE CDS IMPLEMENTATION

4.1 Information to users about the CDS system and its functions is appropriate

☐ Strongly disagree ☐ Disagree ☐ Somewhat disagree ☐ Undecided ☐ Somewhat agree ☐ Agree ☐ Strongly agree

Notes:	Follow-up actions:

Rationale

Clear communication about the CDS intervention, facilitates awareness about the presence of the system and its functions. It may also facilitate more user participation.

CDS training facilitates knowledge about CDS systems and the skills needed to use them. Systems that are designed to be more intuitive may not require any training.

References: [22, 28, 32, 35-37, 39, 44, 47, 51, 62, 63, 66, 67, 69, 71, 73, 74, 81, 96]

How to evaluate

Consider the following questions:

- Is the communication and documentation about the CDS appropriate?
- Are help topics related to the functioning of the CDS system available to users?
- If necessary, is user training available?

Examples

Positive examples could include:

- A communication package is developed to inform healthcare providers about the launch of the CDS.
- Healthcare providers are aware that the system only provides advice for some clinical conditions.
- The users have received a training session in a demo environment to enable them to experience the system's features.
- A user support helpdesk has been installed.

Negative examples could include:

- The system is activated without any communication or information being provided. The users are not aware that a screen providing decision support is available.
- A training session was organised but many of the users did not attend.
- The system is down due to technical problems and the users have not been informed.
- The system provides patient-directed decision support but the patients do not have the required computer skills.

4.2 Other barriers and facilitators to compliance with the decision support advice are assessed/addressed

☐ Strongly disagree ☐ Disagree ☐ Somewhat disagree ☐ Undecided ☐ Somewhat agree ☐ Agree ☐ Strongly agree

Notes:	Follow-up actions:

Rationale

Implementing a CDS intervention may only be a part of the solution for a quality objective. Adherence to specific decision support advice may be affected by a wider range of factors that CDS is not able to address. These factors may be applicable generally, or they may be specific to a local context.

An implementation strategy that assesses the barriers and facilitating factors, and that plans actions accordingly, is likely to improve the success of quality improvement efforts.

References: [22, 29, 31, 35, 37, 39, 42, 45, 47, 49-52, 59, 60, 63, 65, 71-73, 75, 82, 86]

How to evaluate

Consider the following questions:

- Is there an assessment of the beliefs, attitudes and skills of the providers and patients that may affect adherence? Are actions planned/taken accordingly?
- Is there an assessment of the professional interactions affecting adherence, and are actions planned/taken accordingly?
- Is there an assessment of the (dis) incentives affecting the adherence of healthcare providers and patients? Are necessary actions planned/taken?
- Is there an assessment of the issues related to the capacity and resources needed to ensure adherence? Are the necessary actions planned/taken?
- Does the organisational context influence adherence and are actions planned/taken accordingly?
- More specific questions are available in the TICD checklist.[15]

Examples

Positive examples could include:

- The decision support advice is signed by a local opinion leader to help overcome indifference or resistance on the part of the users.
- The new clinical skills that are required to ensure adherence to the recommended action are practised at an educational meeting.
- Adhering to the decision support advice requires more time from the healthcare providers. Therefore the number of targeted recommendations is limited and incentives are provided.

Negative examples could include:

- Multiple guidelines from regional, national or international organisations make conflicting recommendations and this confuses users.
- Every month the healthcare providers receive an automatic list that identifies patients who have gaps in the healthcare they are receiving. However, there is no procedure planned for contacting the patients.

Additional examples are available in the TICD checklist. [15]

4.3 Implementation is stepwise and the improvements in the CDS system are continuous

☐ Strongly disagree ☐ Disagree ☐ Somewhat disagree ☐ Undecided ☐ Somewhat agree ☐ Agree ☐ Strongly agree

Notes:	Follow-up actions:

Rationale

A CDS system is more likely to succeed if it is implemented gradually to full-scale. This is because issues can be identified and addressed at an early stage. Collecting user feedback and monitoring a system's performance and usage provides opportunities to fix malfunctions and to make the system more effective in the long-term.[97] Fixing problems quickly facilitates better usage of the system and can help to prevent frustrated users from quitting.

Monitoring the outcomes of a CDS initiative is an important way to evaluating the impact of the system and to identify important deviations from evidence-based care. Some CDS systems allow clinical quality measures to be reported automatically.[98]

References: [22, 28, 30-35, 37, 44, 45, 47, 51, 57, 62, 63, 73, 77, 83, 84, 99]

How to evaluate

Consider the following questions:

- Is the implementation of the CDS stepwise?
- Is a plan in place to collect user feedback and to monitor system usage, performance and outcomes?
- Are malfunctions and other problems with use of the CDS quickly fixed?

Examples

Positive examples could include:

- The implementation of the system is done in phases, initially using a smaller group of users, then a larger group, and finally a large user group.
- System logs are monitored and end-user input is collected to improve the system continuously.
- The CDS includes an in-built clinical quality-measuring system to report how many patients receive a procedure versus all the patients who are eligible to receive the procedure.

Negative examples could include:

- The usage of CDS is not monitored and those responsible for the system are not aware that there is a problem that is causing low levels of use.
- The reminders in the system contain a dialogue box that allows problems to be communicated, but the users have stopped using it because they have not received any feedback.
- Some decision support is no longer triggered because changes have been made to the clinical classification codes. The decision support logic has not been adapted accordingly.

4.4 Governance of the CDS implementation is appropriate

☐ Strongly disagree ☐ Disagree ☐ Somewhat disagree ☐ Undecided ☐ Somewhat agree ☐ Agree ☐ Strongly agree

Notes:	Follow-up actions:

Rationale

Governance of the implementation is multifaceted, may vary with the setting and the context, and may include:

- Decisions and strategies about prioritised goals;
- Arranging the required budgets and resources;
- Establishing accountability for achieving goals;
- Establishing a knowledge management plan (see also 2.1);
- Collaboration between clinical and technical groups;
- Safeguarding medico-legal aspects surrounding CDS;
- Ensuring that effective procedures for addressing problems are in place (see also 4.3)

Involving all the key stakeholders in the planning, development and management of a CDS intervention helps to ensure that strategy is relevant and that the required level of support is obtained. A multidisciplinary team may be a more effective way of addressing challenges.

How to evaluate

Consider the following questions:

- Are all the key stakeholders involved in the planning and implementation of the system?
- Is the CDS initiative governed in an efficient, sustainable and equitable way?

Good governance requires that CDS is implemented sustainably and equitably with regard to resource-poor communities.[100] Without it, CDS implementation can potentially undermine health equity.

Sustainable governance includes a clear vision of how the CDS will be used in the future, as well as the effort required to add content and other CDS functions. Large organisations may also want to partake in wider networks related to policy and data standards.

References: [22, 28, 31, 32, 34, 35, 37, 39, 44, 47, 49, 62, 63, 77, 82, 87]

Examples

Positive examples could include:

- The CDS intervention is planned in consultation with a multidisciplinary group that includes health service managers, healthcare providers, guideline experts, quality assurance experts, health service IT workers, and the system's developer).
- An internal policy is available which describes the management of the CDS intervention in its entirety.
- Locally developed decision support is successfully transferred for use at the national level.

Negative examples could include:

- The communication between healthcare providers, system developers and implementers is minimal and people have only limited insight about each other's areas of work.
- Economic constraints hinder system updates.
- Due to suboptimal planning, the requested functionalities do not match the needs of the targeted users.

OVERALL CONCLUSION

Notes:

Follow-up actions:

REFERENCES

1. Field MJE, Lohr KNE. Clinical practice guidelines : directions for a new program. Washington, D.C.: National Academy Press; 1990.

2. Lau R, Stevenson F, Ong BN, Dziedzic K, Treweek S, Eldridge S et al. Achieving change in primary care--causes of the evidence to practice gap: systematic reviews of reviews. Implement Sci. 2016;11:40.

3. Scott IA, Glasziou PP. Improving effectiveness of clinical medicine: the need for better translation of science into practice. Med J Aust. 2012;197(7):374-8.

4. Fretheim A, Flottorp S, Oxman A. Effect of Interventions for Implementing Clinical Practice Guidelines. NIPH Systematic Reviews: Executive Summaries. Oslo, Norway2015.

5. Roshanov PS, Fernandes N, Wilczynski JM, Hemens BJ, You JJ, Handler SM et al. Features of effective computerised clinical decision support systems: meta-regression of 162 randomised trials. BMJ. 2013;346:f657.

6. Moja L, Kwag KH, Lytras T, Bertizzolo L, Brandt L, Pecoraro V et al. Effectiveness of computerized decision support systems linked to electronic health records: a systematic review and meta-analysis. Am J Public Health. 2014;104(12):e12-22.

7. Watson R. European Union leads way on e-health, but obstacles remain. BMJ. 2010;341:c5195.

8. Myers RB, Jones SL, Sittig DF. Review of Reported Clinical Information System Adverse Events in US Food and Drug Administration Databases. Appl Clin Inform. 2011;2(1):63-74.

9. Coiera E, Westbrook J, Wyatt J. The safety and quality of decision support systems. Yearb Med Inform. 2006:20-5.

10. Lobach DF. The road to effective clinical decision support: are we there yet? BMJ. 2013;346:f1616.

11. Coiera E, Aarts J, Kulikowski C. The dangerous decade. J Am Med Inform Assoc. 2012;19(1):2-5.

12. Gawande A. The checklist manifesto : how to get things right. 1st ed. New York: Metropolitan Books; 2010.

13. Bodenheimer T, Sinsky C. From triple to quadruple aim: care of the patient requires care of the provider. Ann Fam Med. 2014;12(6):573-6.

14. Prior M, Guerin M, Grimmer-Somers K. The effectiveness of clinical guideline implementation strategies--a synthesis of systematic review findings. J Eval Clin Pract. 2008;14(5):888-97.

15. Flottorp SA, Oxman AD, Krause J, Musila NR, Wensing M, Godycki-Cwirko M et al. A checklist for identifying determinants of practice: a systematic review and synthesis of frameworks and taxonomies of factors that prevent or enable improvements in healthcare professional practice. Implement Sci. 2013;8:35.

16. Andrews J, Guyatt G, Oxman AD, Alderson P, Dahm P, Falck-Ytter Y et al. GRADE guidelines: 14. Going from evidence to recommendations: the significance and presentation of recommendations. J Clin Epidemiol. 2013;66(7):719-25.

17. Atkins D, Best D, Briss PA, Eccles M, Falck-Ytter Y, Flottorp S et al. Grading quality of evidence and strength of recommendations. BMJ. 2004;328(7454):1490.

18. Kawamoto K, Houlihan CA, Balas EA, Lobach DF. Improving clinical practice using clinical decision support systems: a systematic review of trials to identify features critical to success. BMJ. 2005;330(7494):765.

19. Pantoja T, Green ME, Grimshaw J, Denig P, Durieux P, Gill P et al. Manual paper reminders: effects on professional practice and health care outcomes. Cochrane Database of Systematic Reviews. 2014(9):CD001174.

20. Brouwers MC, Kho ME, Browman GP, Burgers JS, Cluzeau F, Feder G et al. AGREE II: advancing guideline development, reporting, and evaluation in health care. Prev Med. 2010;51(5):421-4.

21. Shiffman RN, Dixon J, Brandt C, Essaihi A, Hsiao A, Michel G et al. The GuideLine Implementability Appraisal (GLIA): development of an instrument to identify obstacles to guideline implementation. BMC Med Inform Decis Mak. 2005;5:23.

22. Osheroff JA, Teich JM, Levick D, Saldana L, Velasco TF, Sittig DF et al. Improving outcomes with clinical decision support : an implementer's guide. 2nd ed. Chicago, IL: HIMSS; 2012.

23. Fixsen DL, Blase KA, Duda MA, Naoom SF, Van Dyke M. Implementation of evidence-based treatments for children and adolescents: Research findings and their implications for the future. In: Kazdin JRWAE, editor. Evidence-based psychotherapies for children and adolescents, 2nd ed. New York, NY, US: Guilford Press; 2010. p. 435-50.

24. Balshem H, Helfand M, Schunemann HJ, Oxman AD, Kunz R, Brozek J et al. GRADE guidelines: 3. Rating the quality of evidence. J Clin Epidemiol. 2011;64(4):401-6.

25. Van de Velde S, Roshanov P, Heselmans A, Delvaux N, Brandt L, Cloetens H et al. A systematic review of trials evaluating success

features of computerised clinical decision support systems. PROSPERO International prospective register of systematic reviews. 2016.

26. Toolkit GIN PUBLIC. Patient and public involvement in guidelines. Guidelines International Network. 2012.

27. Van de Velde S, Roshanov P, Kortteisto T, Kunnamo I, Aertgeerts B, Vandvik PO et al. Tailoring implementation strategies for evidence-based recommendations using computerised clinical decision support systems: protocol for the development of the GUIDES tools. Implement Sci. 2016;11(1):29.

28. Ash JS, Sittig DF, Guappone KP, Dykstra RH, Richardson J, Wright A et al. Recommended practices for computerized clinical decision support and knowledge management in community settings: a qualitative study. BMC medical informatics and decision making. 2012;12:6.

29. Berlin A, Sorani M, Sim I. A taxonomic description of computer-based clinical decision support systems. Journal of Biomedical Informatics. 2006;39(6):656-67.

30. Handler JA, Feied CF, Coonan K, Vozenilek J, Gillam M, Peacock PR, Jr. et al. Computerized physician order entry and online decision support. Academic emergency medicine : official journal of the Society for Academic Emergency Medicine. 2004;11(11):1135-41.

31. Horsky J, Schiff GD, Johnston D, Mercincavage L, Bell D, Middleton B. Interface design principles for usable decision support: a targeted review of best practices for clinical prescribing interventions. Journal of Biomedical Informatics. 2012;45(6):1202-16.

32. Improving Medication Use and Outcomes with Clinical Decision Support: A Step-by-Step Guide. Osheroff JA, ed. 2009. Healthcare Information Management and Systems Society.

33. Sweidan M, Williamson M, Reeve JF, Harvey K, O'Neill JA, Schattner P et al. Identification of features of electronic prescribing systems to support quality and safety in primary care using a modified Delphi process. BMC medical informatics and decision making. 2010;10(1):21.

34. Teich JM, Osheroff JA, Pifer EA, Sittig DF, Jenders RA, Panel CDSER. Clinical decision support in electronic prescribing: recommendations and an action plan: report of the joint clinical decision support workgroup. Journal of the American Medical Informatics Association : JAMIA. 2005;12(4):365-76.

35. Wright A, Ash JS, Erickson JL, Wasserman J, Bunce A, Stanescu A et al. A qualitative study of the activities performed by people involved in clinical decision support: recommended practices for success. Journal of the American Medical Informatics Association. 2014;21(3):464-72.

36. Kilsdonk E, Peute LW, Jaspers MW. Factors influencing implementation success of guideline-based clinical decision support systems: A systematic review and gaps analysis. Int J Med Inform. 2017;98:56-64.

37. Sittig DF, Singh H. A new sociotechnical model for studying health information technology in complex adaptive healthcare systems. Qual Saf Health Care. 2010;19 Suppl 3:i68-74.

38. Arditi C, Rege-Walther M, Wyatt JC, Durieux P, Burnand B. Computer-generated reminders delivered on paper to healthcare professionals: effects on professional practice and health care outcomes. Cochrane Database Syst Rev. 2012;12:CD001175.

39. Damiani G, Pinnarelli L, Colosimo SC, Almiento R, Sicuro L, Galasso R et al. The effectiveness of computerized clinical guidelines in the process of care: a systematic review. BMC health services research. 2010;10(1):2.

40. Fillmore CL, Bray BE, Kawamoto K. Systematic review of clinical decision support interventions with potential for inpatient cost reduction. BMC medical informatics and decision making. 2013;13(1):135.

41. Gillaizeau F, Chan E, Trinquart L, Colombet I, Walton RT, Rege-Walther M et al. Computerized advice on drug dosage to improve prescribing practice. Cochrane Database Syst Rev. 2013;11:CD002894.

42. Goldzweig CL, Orshansky G, Paige NM, Miake-Lye IM, Beroes JM, Ewing BA et al. Electronic Health Record-Based Interventions for Improving Appropriate Diagnostic Imaging: A Systematic Review and Meta-analysis. Annals of internal medicine. 2015;162(8):557-65.

43. Holt TA, Thorogood M, Griffiths F. Changing clinical practice through patient specific reminders available at the time of the clinical encounter: systematic review and meta-analysis. Journal of General Internal Medicine. 2012;27(8):974-84.

44. Mollon B, Chong JJR, Holbrook AM, Sung M, Thabane L, Foster G. Features predicting the success of computerized decision support for prescribing: a systematic review of randomized controlled trials. BMC medical informatics and decision making. 2009;9(1):11.

45. Nuckols TK, Smith-Spangler C, Morton SC, Asch SM, Patel VM, Anderson LJ et al. The effectiveness of computerized order entry at reducing preventable adverse drug events and medication errors in hospital settings: a systematic review and meta-analysis. Systematic reviews. 2014;3(1):56.

46. Pearson S-A, Moxey A, Robertson J, Hains I, Williamson M, Reeve J et al. Do computerised clinical decision support systems for prescribing change practice? A systematic review of the literature (1990-2007). BMC health services research. 2009;9:154.

47. Roshanov PS, Fernandes N, Wilczynski JM, Hemens BJ, You JJ, Handler SM et al. Features of effective computerised clinical decision support systems: meta-regression of 162 randomised trials. BMJ. 2013;346(f657).

48. Schedlbauer A, Prasad V, Mulvaney C, Phansalkar S, Stanton W, Bates DW et al. What evidence supports the use of computerized alerts and prompts to improve clinicians' prescribing behavior? Journal of the American Medical Informatics Association : JAMIA. 2009;16(4):531-8.

49. Shojania KG, Jennings A, Mayhew A, Ramsay C, Eccles M, Grimshaw J. Effect of point-of-care computer reminders on physician behaviour: a systematic review. CMAJ : Canadian Medical Association journal = journal de l'Association medicale canadienne. 2010;182(5):E216-25.

50. Goud R, van Engen-Verheul M, de Keizer NF, Bal R, Hasman A, Hellemans IM et al. The effect of computerized decision support on barriers to guideline implementation: A qualitative study in outpatient cardiac rehabilitation. International Journal of Medical

Informatics. 2010;79(6):430-7.

51. Moxey A, Robertson J, Newby D, Hains I, Williamson M, Pearson S-A. Computerized clinical decision support for prescribing: provision does not guarantee uptake. Journal of the American Medical Informatics Association : JAMIA. 2010;17(1):25-33.

52. Sequist TD, Gandhi TK, Karson AS, Fiskio JM, Bugbee D, Sperling M et al. A randomized trial of electronic clinical reminders to improve quality of care for diabetes and coronary artery disease. Journal of the American Medical Informatics Association : JAMIA. 2005;12(4):431-7.

53. McCormack JL, Ash JS. Clinician perspectives on the quality of patient data used for clinical decision support: a qualitative study. AMIA Annual Symposium proceedings / AMIA Symposium AMIA Symposium. 2012;2012:1302-9.

54. Sittig DF, Wright A, Osheroff JA, Middleton B, Teich JM, Ash JS et al. Grand challenges in clinical decision support. J Biomed Inform. 2008;41(2):387-92.

55. van der Sijs H, Aarts J, Vulto A, Berg M. Overriding of drug safety alerts in computerized physician order entry. J Am Med Inform Assoc. 2006;13(2):138-47.

56. Ahmadian L, van Engen-Verheul M, Bakhshi-Raiez F, Peek N, Cornet R, de Keizer NF. The role of standardized data and terminological systems in computerized clinical decision support systems: literature review and survey. International Journal of Medical Informatics. 2011;80(2):81-93.

57. Martens JD, van der Aa A, Panis B, van der Weijden T, Winkens RA, Severens JL. Design and evaluation of a computer reminder system to improve prescribing behaviour of GPs. Studies in health technology and informatics. 2006;124:617-23.

58. Miller A, Moon B, Anders S, Walden R, Brown S, Montella D. Integrating computerized clinical decision support systems into clinical work: A meta-synthesis of qualitative research. Int J Med Inform. 2015;84(12):1009-18.

59. Bindels R, Hasman A, Derickx M, van Wersch JWJ, Winkens RAG. User satisfaction with a real-time automated feedback system for general practitioners: a quantitative and qualitative study. International Journal for Quality in Health Care. 2003;15(6):501-8.

60. Bindels R, Hasman A, van Wersch JW, Talmon J, Winkens RA. Evaluation of an automated test ordering and feedback system for general practitioners in daily practice. Int J Med Inform. 2004;73(9-10):705-12.

61. Hobbs FD, Delaney BC, Carson A, Kenkre JE. A prospective controlled trial of computerized decision support for lipid management in primary care. Family Practice. 1996;13(2):133-7.

62. Coleman J, Slee A. Guidelines for Hazard Review of ePrescribing Decision Support. 2009.

63. Kilsdonk E, Peute LWP, Knijnenburg SL, Jaspers MWM. Factors known to influence acceptance of clinical decision support systems. Studies in health technology and informatics. 2011;169:150-4.

64. Main C, Moxham T, Wyatt JC, Kay J, Anderson R, Stein K. Computerised decision support systems in order communication for diagnostic, screening or monitoring test ordering: systematic reviews of the effects and cost-effectiveness of systems. Health technology assessment (Winchester, England). 2010;14(48):1-227.

65. Ackerman SL, Gonzales R, Stahl MS, Metlay JP. One size does not fit all: evaluating an intervention to reduce antibiotic prescribing for acute bronchitis. BMC health services research. 2013;13(1):462.

66. Lugtenberg M, Weenink JW, van der Weijden T, Westert GP, Kool RB. Implementation of multiple-domain covering computerized decision support systems in primary care: a focus group study on perceived barriers. BMC Med Inform Decis Mak. 2015;15:82.

67. McDermott L, Yardley L, Little P, van Staa T, Dregan A, McCann G et al. Process evaluation of a point-of-care cluster randomised trial using a computer-delivered intervention to reduce antibiotic prescribing in primary care. BMC Health Serv Res. 2014;14:594.

68. Apkon M, Mattera JA, Lin Z, Herrin J, Bradley EH, Carbone M et al. A randomized outpatient trial of a decision-support information technology tool. Archives of Internal Medicine. 2005;165(20):2388-94.

69. Barnard KD, Cradock S, Parkin T, Skinner TC. Effectiveness of a computerised assessment tool to prompt individuals with diabetes to be more active in consultations. Practical Diabetes International. 2007;24(1):36-41.

70. Bloomfield HE, Nelson DB, van Ryn M, Neil BJ, Koets NJ, Basile JN et al. A trial of education, prompts, and opinion leaders to improve prescription of lipid modifying therapy by primary care physicians for patients with ischemic heart disease. Quality & safety in health care. 2005;14(4):258-63.

71. Flottorp S, Havelsrud K, Oxman AD. Process evaluation of a cluster randomized trial of tailored interventions to implement guidelines in primary care--why is it so hard to change practice? Family Practice. 2003;20(3):333-9.

72. Fretheim A, Havelsrud K, Oxman A. Rational Prescribing in Primary care (RaPP): process evaluation of an intervention to improve prescribing of antihypertensive and cholesterol-lowering drugs. Implementation Science. 2006;1(1):19.

73. Hetlevik I, Holmen J, Krüger O, Kristensen P, Iversen H. Implementing clinical guidelines in the treatment of hypertension in general practice. Blood pressure. 1998;7(5-6):270-6.

74. Lugtenberg M, Pasveer D, van der Weijden T, Westert GP, Kool RB. Exposure to and experiences with a computerized decision support intervention in primary care: results from a process evaluation. BMC Fam Pract. 2015;16(1):141.

75. Roumie CL, Elasy TA, Wallston KA, Pratt S, Greevy RA, Liu X et al. Clinical inertia: a common barrier to changing provider prescribing behavior. Joint Commission journal on quality and patient safety / Joint Commission Resources. 2007;33(5):277-85.

76. Tierney WM, Miller ME, Overhage JM, McDonald CJ. Physician inpatient order writing on microcomputer workstations. Effects on resource utilization. JAMA : the journal of the American Medical Association. 1993;269(3):379-83.

77. Wright A, Sittig DF, Ash JS, Bates DW, Feblowitz J, Fraser G et al. Governance for clinical decision support: case studies and recommended practices from leading institutions. J Am Med Inform Assoc. 2011;18(2):187-94.

78. McCowan C, Neville RG, Ricketts IW, Warner FC, Hoskins G, Thomas GE. Lessons from a randomized controlled trial designed to evaluate computer decision support software to improve the management of asthma. Medical Informatics and the Internet in Medicine. 2001;26(3):191-201.

79. Qaseem A, Forland F, Macbeth F, Ollenschlager G, Phillips S, van der Wees P et al. Guidelines International Network: toward international standards for clinical practice guidelines. Ann Intern Med. 2012;156(7):525-31.

80. Elwyn G, O'Connor A, Stacey D, Volk R, Edwards A, Coulter A et al. Developing a quality criteria framework for patient decision aids: online international Delphi consensus process. BMJ. 2006;333(7565):417.

81. Goddard K, Roudsari A, Wyatt JC. Automation bias: a systematic review of frequency, effect mediators, and mitigators. Journal of the American Medical Informatics Association. 2012;19(1):121-7.

82. Lobach D, Sanders GD, Bright TJ, Wong A, Dhurjati R, Bristow E et al. Enabling health care decisionmaking through clinical decision support and knowledge management. Evidence report/technology assessment. 2012(203):1-784.

83. Bates DW, Kuperman GJ, Wang S, Gandhi T, Kittler A, Volk L et al. Ten commandments for effective clinical decision support: making the practice of evidence-based medicine a reality. J Am Med Inform Assoc. 2003;10(6):523-30.

84. Payne TH, Hines LE, Chan RC, Hartman S, Kapusnik-Uner J, Russ AL et al. Recommendations to improve the usability of drug-drug interaction clinical decision support alerts. J Am Med Inform Assoc. 2015;22(6):1243-50.

85. Pelayo S, Marcilly R, Bernonville S, Leroy N, Beuscart-Zephir M-C. Human factors based recommendations for the design of medication related clinical decision support systems (CDSS). Studies in health technology and informatics. 2011;169:412-6.

86. Keeffe B, Subramanian U, Tierney WM, Udris E, Willems J, McDonell M et al. Provider response to computer-based care suggestions for chronic heart failure. Medical Care. 2005;43(5):461-5.

87. Jenders RA. Standards in health information technology: promise and challenges. AMIA Annual Symposium proceedings / AMIA Symposium AMIA Symposium. 2007:1179-80.

88. DIS-ISO. 9241-210: 2010. Ergonomics of human system interaction-Part 210: Human-centred design for interactive systems. International Standardization Organization (ISO) Switzerland. 2009.

89. ISO. IEC25010: 2011 Systems and software engineering–Systems and software Quality Requirements and Evaluation (SQuaRE)–System and software quality models. International Organization for Standardization. 2011;34:2910.

90. Cowan JA, Heckerling PS, Parker JB. Effect of a fact sheet reminder on performance of the periodic health examination: a randomized controlled trial. American journal of preventive medicine. 1992;8(2):104-9.

91. Rotman BL, Sullivan AN, McDonald TW, Brown BW, DeSmedt P, Goodnature D et al. A randomized controlled trial of a computer-based physician workstation in an outpatient setting: implementation barriers to outcome evaluation. J Am Med Inform Assoc. 1996;3(5):340-8.

92. Brandt L, Vandvik PO, Alonso-Coello P, Akl EA, Thornton J, Rigau D et al. Multilayered and digitally structured presentation formats of trustworthy recommendations: a combined survey and randomised trial. BMJ Open. 2017;7(2):e011569.

93. Semler MW, Weavind L, Hooper MH, Rice TW, Gowda SS, Nadas A et al. An Electronic Tool for the Evaluation and Treatment of Sepsis in the ICU: A Randomized Controlled Trial. Critical Care Medicine. 2015;43(8):1595-602.

94. Bates DW, Evans RS, Murff H, Stetson PD, Pizziferri L, Hripcsak G. Detecting adverse events using information technology. Journal of the American Medical Informatics Association : JAMIA. 2003;10(2):115-28.

95. Vervloet M, Linn AJ, van Weert JCM, de Bakker DH, Bouvy ML, van Dijk L. The effectiveness of interventions using electronic reminders to improve adherence to chronic medication: a systematic review of the literature. Journal of the American Medical Informatics Association : JAMIA. 2012;19(5):696-704.

96. El-Kareh RE, Gandhi TK, Poon EG, Newmark LP, Ungar J, Orav EJ et al. Actionable reminders did not improve performance over passive reminders for overdue tests in the primary care setting. J Am Med Inform Assoc. 2011;18(2):160-3.

97. Wright A, Hickman TT, McEvoy D, Aaron S, Ai A, Andersen JM et al. Analysis of clinical decision support system malfunctions: a case series and survey. J Am Med Inform Assoc. 2016;23(6):1068-76.

98. Kukhareva PV, Kawamoto K, Shields DE, Barfuss DT, Halley AM, Tippetts TJ et al. Clinical Decision Support-based Quality Measurement (CDS-QM) Framework: Prototype Implementation, Evaluation, and Future Directions. AMIA Annu Symp Proc. 2014;2014:825-34.

99. Jenders RA, Osheroff JA, Sittig DF, Pifer EA, Teich JM. Recommendations for clinical decision support deployment: synthesis of a roundtable of medical directors of information systems. AMIA Annu Symp Proc. 2007:359-63.

100. Fong H, Harris E. Technology, innovation and health equity. Bull World Health Organ. 2015;93(7):438-A.

ANNEX 1: OVERVIEW OF CHECKLIST FACTORS AND 'HOW-TO-EVALUATE' QUESTIONS.

	Disagree Strongly	Disagree	Somewhat disagree	Undecided	Somewhat agree	Agree	Strongly agree
**Domain 1: The CDS context**							
**1.1. CDS can achieve the planned quality objectives**<br>• Does CDS address the factors that explain the current behaviour of healthcare providers and patients?<br>• Does the available evidence support the use of CDS for the given outcomes, tasks and settings?	☐	☐	☐	☐	☐	☐	☐
**1.2 The quality of the patient data is sufficient**<br>• Is the structured patient data that is needed to achieve the CDS objective sufficiently accurate and complete to allow the use of CDS?<br>• If necessary, can the quality of the data be improved or can the CDS itself improve the data quality?	☐	☐	☐	☐	☐	☐	☐
**1.3 Stakeholders and users accept CDS**<br>• Is there a clear benefit to the users who will engage with the CDS?<br>• Do the users and stakeholders have a positive attitude towards the use of CDS?<br>• If necessary, is it possible to increase user and stakeholder acceptance?	☐	☐	☐	☐	☐	☐	☐
**1.4 CDS can be added to the existing workload, workflows and systems**<br>• Is the required hardware available and what will the impact be of adding CDS to the existing information systems?<br>• Is it feasible to introduce CDS, given the current workload and the usual work processes?<br>• If necessary, can the workload or the work processes be changed or can the CDS system improve the workload or work processes?	☐	☐	☐	☐	☐	☐	☐
**Domain 2: The CDS content**							
**2.1 The content provides trustworthy evidence-based information**<br>• Do the organisation(s) and people that developed the decision support have credibility?<br>• Is the advice supported by up-to-date scientific evidence and is the type and quality of this evidence clear to the user?<br>• Is the decision support clear on the benefits and harms of the different management options?	☐	☐	☐	☐	☐	☐	☐
**2.2 The decision support is relevant and accurate**<br>• Does the decision support contain accurate information that is pertinent to the care of the patient?<br>• Does the decision support address the information needs of the users?<br>• Is it clear to the users why the decision support information is provided for a given patient?	☐	☐	☐	☐	☐	☐	☐
**2.3 The decision support provides an appropriate call to action**<br>• Is the recommended action clear enough for the targeted users to act on?<br>• Is the clinical importance and urgency of the recommended action sufficiently clear?<br>• Is the advice applicable in the setting in which it will be implemented?<br>• Is it clear how the recommended action fits with other current guidelines?	☐	☐	☐	☐	☐	☐	☐
**2.4 The amount of decision support is manageable for the target user**<br>• Is the total amount of decision support manageable for the healthcare provider?<br>• Is the amount of decision support per patient manageable?	☐	☐	☐	☐	☐	☐	☐

	Disagree Strongly	Disagree	Somewhat disagree	Undecided	Somewhat agree	Agree	Strongly agree
**Domain 3: The CDS system**							
**3.1 The system is easy to use**<br>• Is it easy for users to interact with the CDS system?<br>• Does the system facilitate (or, at least, not hinder) the workflow of the healthcare providers?<br>• Can the system be customised to provide better user support<br>• Is the system always up and running?	☐	☐	☐	☐	☐	☐	☐
**3.2 The decision support is well delivered**<br>• Is the advice delivered in an appropriate mode, format and channel?<br>• Is the display of the decision support eye-catching, intuitive, concise, consistent and unambiguous?<br>• Is it appropriate to use specific functions (e.g. pop-ups, computerised restrictions, indications of (dis)agreement) for prioritised decision support?	☐	☐	☐	☐	☐	☐	☐
**3.3 The system delivers the decision support to the right target person**<br>• Is the system reaching the targeted users (healthcare providers and/or patients)?<br>• Is the system able to facilitate team processes when these are needed?	☐	☐	☐	☐	☐	☐	☐
**3.4 The decision support is available at the right time**<br>• Does the system provide the decision support at a moment of need?	☐	☐	☐	☐	☐	☐	☐
**Domain 4: The CDS implementation**							
**4.1 Information to users about the CDS and its functions is appropriate**<br>• Is the communication and documentation about the CDS appropriate?<br>• Are help topics related to the functioning of the CDS system available to users?<br>• If necessary, is user training available?	☐	☐	☐	☐	☐	☐	☐
**4.2 Other barriers and facilitators to compliance with the decision support advice are assessed/addressed**<br>• Is there an assessment of the beliefs, attitudes and skills of the providers and patients that may affect adherence? Are actions planned/taken accordingly?<br>• Is there an assessment of the professional interactions affecting adherence, and are actions planned/taken accordingly?<br>• Is there an assessment of the (dis)incentives affecting the adherence of healthcare providers and patients? Are necessary actions planned/taken?<br>• Is there an assessment of the issues related to the capacity and resources needed to ensure adherence? Are the necessary actions planned/taken?<br>• Does the organisational context influence adherence and are actions planned/taken accordingly?	☐	☐	☐	☐	☐	☐	☐
**4.3 Implementation is stepwise and the improvements in the CDS system are continuous**<br>• Is the implementation of the CDS stepwise?<br>• Is a plan in place to collect user feedback and to monitor system usage, performance and outcomes?<br>• Are malfunctions and other problems with use of the CDS quickly fixed?	☐	☐	☐	☐	☐	☐	☐
**4.4 Governance of the CDS implementation is appropriate**<br>• Are all the key stakeholders involved in the planning and implementation of the system?<br>• Is the CDS initiative governed in an efficient, sustainable and equitable way?	☐	☐	☐	☐	☐	☐	☐

OVERALL CONCLUSION

Notes:	Follow-up actions:
