# Supplementary material for: The GUIDES checklist: development of a tool to improve the successful use of guideline-based computerised clinical decision support
Source: Implement Sci. 2018 Jun 25;13:86. doi: 10.1186/s13012-018-0772-3 (PMC6019508; doi:10.1186/s13012-018-0772-3)
Supplement: Supplementary file 4 — Importance ratings of GUIDES factors. (DOCX 28 kb) [file 13012_2018_772_MOESM4_ESM.docx]

# Additional file 4

# Expert panel feedback about the importance of the GUIDES factors

**1. Are these factors important for the success of guideline-based CDS interventions in general?**

|  | **Always or almost always important** | **Sometimes important** | **Never or almost never important** | **I don't know** |
| --- | --- | --- | --- | --- |
| **1.1 CDS can adequately address the needs and objectives** | 39 | 4 | 1 | 1 |
| **1.2 The quality of the patient data is adequate** | 38 | 7 | 0 | 0 |
| **1.3 Stakeholders and users accept CDS** | 36 | 8 | 0 | 0 |
| **1.4 CDS can be added to the existing workload and workflows** | 38 | 7 | 0 | 0 |
| **2.1 The content is high quality and trustworthy** | 43 | 2 | 0 | 0 |
| **2.2 The content is relevant and accurate** | 44 | 0 | 0 | 0 |
| **2.3 The decision support provides an adequate call to action** | 32 | 12 | 0 | 0 |
| **2.4 The amount of decision support is manageable** | 37 | 6 | 0 | 1 |
| **3.1 The system is easy to use** | 38 | 6 | 0 | 0 |
| **3.2 The delivery of the decision support is appropriate** | 37 | 7 | 0 | 0 |
| **3.3 The decision support is directed at the right person** | 42 | 1 | 0 | 1 |
| **3.4 The decision support comes at the right time** | 39 | 4 | 1 | 0 |
| **3.5 The display of the decision support is adequate** | 33 | 11 | 0 | 0 |
| **3.6 Critical decision support is difficult to overlook or neglect** | 33 | 9 | 0 | 2 |
| **4.1 Access to the system is easy** | 38 | 6 | 0 | 0 |
| **4.2 Information about the CDS and its functions is adequate** | 27 | 15 | 1 | 1 |
| **4.3 Factors that influence adherence to the decision support advice are addressed** | 30 | 13 | 0 | 1 |
| **4.4 Implementation is stepwise and system improvement is continual** | 30 | 13 | 1 | 0 |
| **4.5 Governance is adequate** | 31 | 13 | 0 | 0 |
| **N=45** |  |  |  |  |

**2. In the following list of 16 important factors for succesful guideline-based CDS interventions, please select the five that are most important to you and rank them in order of importance. Choice 1 should be the most important factor and Choice 5 the fifth most important factor.**

|  | **Choice 1** | **Choice 2** | **Choice 3** | **Choice 4** | **Choice 5** | **Total score** |
| --- | --- | --- | --- | --- | --- | --- |
| **1.1 CDS can achieve the defined quality objectives** | 10 | 2 | 1 | 1 | 1 | 64 |
| **1.2 The quality of the patient data is adequate** | 3 | 4 | 3 | 2 | 3 | 47 |
| **1.3 Stakeholders and users accept CDS** | 3 | 3 | 3 | 2 | 3 | 43 |
| **1.4 CDS can be added to the existing workload, workflows and systems** | 5 | 4 | 7 | 3 | 3 | 71 |
| **2.1 The content provides trustworthy evidence-based information** | 10 | 10 | 4 | 1 | 2 | 106 |
| **2.2 The content is relevant and accurate** | 6 | 9 | 2 | 2 | 0 | 76 |
| **2.3 The decision support provides an appropriate call to action** | 0 | 2 | 4 | 3 | 2 | 28 |
| **2.4 The amount of decision support is manageable for the target user** | 0 | 0 | 2 | 1 | 1 | 9 |
| **3.1 The system is easy to use** | 2 | 1 | 4 | 8 | 2 | 44 |
| **3.2 The decision support is well delivered** | 0 | 0 | 2 | 2 | 3 | 13 |
| **3.3 The system delivers the decision support to the right target person** | 0 | 0 | 4 | 2 | 4 | 23 |
| **3.4 The decision support is available at the right time** | 0 | 2 | 0 | 7 | 2 | 24 |
| **4.1 Information to users about the CDS system and its functions is appropriate** | 0 | 0 | 0 | 1 | 0 | 2 |
| **4.2 Other barriers and facilitators to compliance with the decision support advice are assessed/addressed** | 0 | 0 | 0 | 1 | 5 | 7 |
| **4.3 Implementation is stepwise and the improvements in the CDS system are continuous** | 1 | 1 | 0 | 3 | 4 | 19 |
| **4.4 Governance of the CDS implementation is appropriate** | 0 | 1 | 2 | 0 | 2 | 12 |
| **N=39** |  |  |  |  |  |  |
